# A Polymeric Nanoparticle Formulation for Targeted mRNA Delivery to Fibroblasts

**DOI:** 10.1002/advs.202205475

**Published:** 2022-12-18

**Authors:** Artur Filipe Rodrigues, Catarina Rebelo, Susana Simões, Cristiana Paulo, Sónia Pinho, Vítor Francisco, Lino Ferreira

**Affiliations:** ^1^ CNC–Center for Neurosciences and Cell Biology University of Coimbra Coimbra 3000‐517 Portugal; ^2^ Faculty of Medicine Pólo das Ciências da Saúde Unidade Central University of Coimbra Coimbra 3000‐354 Portugal

**Keywords:** CRISPR/Cas9, gene edition, high‐throughput screening, messenger RNA, polymeric nanoparticles

## Abstract

Messenger RNA (mRNA)‐based therapies offer enhanced control over the production of therapeutic proteins for many diseases. Their clinical implementation warrants formulations capable of delivering them safely and effectively to target sites. Owing to their chemical versatility, polymeric nanoparticles can be designed by combinatorial synthesis of different ionizable, cationic, and aromatic moieties to modulate cell targeting, using inexpensive formulation steps. Herein, 152 formulations are evaluated by high‐throughput screening using a reporter fibroblast model sensitive to functional delivery of mRNA encoding Cre recombinase. Using in vitro and in vivo models, a polymeric nanoformulation based on the combination of 3 specific monomers is identified to transfect fibroblasts much more effectively than other cell types populating the skin, with superior performance than lipid‐based transfection agents in the delivery of Cas9 mRNA and guide RNA. This tropism can be explained by receptor‐mediated endocytosis, involving CD26 and FAP, which are overexpressed in profibrotic fibroblasts. Structure‐activity analysis reveals that efficient mRNA delivery required the combination of high buffering capacity and low mRNA binding affinity for rapid release upon endosomal escape. These results highlight the use of high‐throughput screening to rapidly identify chemical features towards the design of highly efficient mRNA delivery systems targeting fibrotic diseases.

## Graphical abstract

Messenger RNA (mRNA) is a promising tool to deliver therapeutic proteins in many diseases, including fibrotic disorders. Here, a polymeric nanoparticle formulation is developed after high‐throughput screening of 152 polymers in a fibroblast model. Using cellular and animal models, this formulation reveals superior performance than lipid‐based transfection agents and targets specificity towards skin fibroblasts.

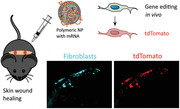

## 1 Introduction

RNA‐based therapies have emerged as promising strategies to regulate cell activity in disease and tissue regeneration.^[^ ^1^ ^]^ Messenger RNA (mRNA)‐based therapeutics offer several advantages relative to plasmid DNA, since mRNA does not require translocation to the nucleus and is more readily biodegradable, thus enabling controlled protein expression without concerns of mutating the genome.^[^ ^2^ ^]^ Transient protein expression could further expand the therapeutic application of RNA‐based therapies to protein replacement and gene editing therapies,^[^ ^2a^ ^]^ which are expected to emerge in the near future. Progress has been made in viral and non‐viral delivery formulations of mRNA‐based therapeutics by maximizing its stability and translation, preventing its immunogenicity, and improving its in vivo delivery.^[^ ^2^ ^]^ However, targeting these formulations to specific cells in the body constitutes an important hurdle in the clinical translation of mRNA‐based therapies, because this is critical to decrease their side effects and maximize their efficacy.

Viral delivery systems have shown good efficiency in mRNA delivery but their limited cargo size, immunogenicity, and safety issues related to off‐target transfection remain important limitations for their broad use, particularly in gene editing.^[^ ^3^ ^]^ Lipid and polymeric nanoparticles (NPs) may be an alternative to viral delivery of mRNA, following their extensive demonstration in the delivery of DNA and short interfering RNAs.^[^ ^2^, ^4^ ^]^ These formulations can protect nucleic acids from degradation and prevent unwanted immune responses, and their physicochemical properties can be readily tuned to control their interaction with cells. The recent clinical approval of lipid nanoparticle (LNP) formulations for the delivery of RNA has encouraged the development of NPs for nucleic acid therapies.^[^ ^5^ ^]^ Yet, their composition can substantially affect its immunogenicity, which is an important aspect considering that inflammatory responses may significantly hamper the translation of mRNA delivered by LNPs.^[^ ^6^ ^]^ Moreover, therapeutic application of LNPs has been limited due to their preferential uptake by hepatocytes in the liver.^[^ ^7^ ^]^ In order to address this challenge, LNPs can be functionalized with targeting antibodies to achieve cellular specificity.^[^ ^8^ ^]^ Although these LNPs have demonstrated therapeutic potential, antibody functionalization requires complex formulation procedures which may deter clinical application.^[^ ^9^ ^]^ Alternatively, recent studies have demonstrated that the biophysical properties of LNPs can be engineered in order to redirect their biodistribution to other cell types (e.g., endothelial and immune cells) in organs such as spleen, kidney, or lung.^[^ ^10^ ^]^ Although the combination of ionizable and cationic moieties was considered essential to modulate tissue tropism, these efforts have failed to target specific cell types.

Polymeric NPs are also attractive candidates for mRNA delivery owing to their greater chemical versatility, resulting from the combination of different amines and divinyl monomers to generate polymer libraries that can be readily synthesized via Michael‐type addition.^[^ ^11^ ^]^ These libraries have identified chemical features and established design principles for electrostatic complexation and delivery of nucleic acids. In the last 5 years, several polymeric NPs have been reported for the delivery of mRNA encoding luciferase, erythropoietin, tumor‐suppressing genes, and gene editing enzymes such as Cre recombinase,^[^ ^12^ ^]^ albeit without specific cell targeting properties. We have previously explored the chemical diversity of combinatorial polymer libraries to design NP formulations for controlled small interfering RNA (siRNA) delivery in vitro and in vivo to the skin.^[^ ^13^ ^]^ The influence of polymer chemistry on NP uptake and bioactivity for siRNA delivery encouraged us to further explore these polymers for mRNA delivery. Nevertheless, NPs developed for siRNA delivery may not be suitable for mRNA delivery.^[^ ^14^ ^]^ While siRNA is typically comprised of two complementary strands of ≈20 nucleotides, mRNA is a single strand spanning up to several kilobases of free nitrogenous bases, which can mediate hydrogen bonding during complexation with cationic NPs, in addition to electrostatic interactions.^[^ ^15^ ^]^

We hypothesized that cellular and tissue tropism of polymeric NPs can be achieved by the combination of specific monomers, without requiring the addition of targeting ligands. To demonstrate this hypothesis, we have prepared a combinatorial library of polymers by Michael‐type addition of amines and divinyl monomers^[^ ^13^, ^16^ ^]^ presenting physicochemical diversity, in order to identify formulations able to interact more specifically with fibroblasts, selected here as the cell target due to their involvement in several diseases.^[^ ^17^ ^]^ These combinations may contain ionizable and cationic moieties to enable electrostatic complexation with nucleic acids and promote endolysosomal escape, while hydrophobic moieties including aromatic compounds improve NP stability in physiological milieu.^[^ ^18^ ^]^ Functional mRNA delivery was evaluated by high‐throughput screening of NP formulations prepared by electrostatic complexation of these polymers with mRNA encoding Cre recombinase, using a fibroblast reporter cell model sensitive to the enzymatic activity of the translated protein. The best polymer candidates were then validated against other skin cell types (keratinocytes, monocytes, and endothelial cells), in order to confirm their tropism for fibroblasts. Polymeric NPs were formulated using three different mRNA molecules: i) one mRNA encoding green fluorescent protein (GFP); and ii) two mRNAs encoding gene editing enzymes (Cre recombinase and Cas9 nuclease). Cre recombinase has been used for more than 30 years in the generation of transgenic animal models with high relevance in fundamental biology,^[^ ^19^ ^]^ whereas Cas9 has revolutionized gene therapy owing to the precise manipulation of genomes using clustered regularly interspaced short palindromic repeats (CRISPR) to correct disease‐causing mutations.^[^ ^3a^ ^]^ The performance of the best formulation was then tested regarding its efficacy and cellular specificity using an in vivo model sensitive to Cre recombinase. Finally, the NPs were characterized for their size, surface charge, cellular internalization, and protein‐encoding activity, in order to establish structure‐activity relationships and understand which physicochemical properties drive mRNA delivery.

## 2 Results and Discussion

### 2.1 High‐Throughput Screening of Polymer Library

**Figure 1:**
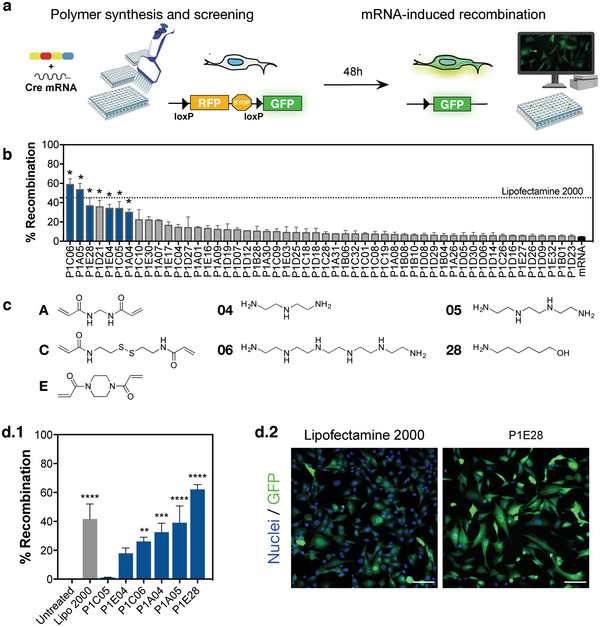
High‐throughput screening of polymer library for mRNA delivery. a) NPs were generated after complexation of mRNA with polymers constituted by a fixed diacrylate moiety (P1) with varying bisacrylamides (A–E) and amines (1–32). Polymer library was screened for the delivery of mRNA encoding Cre recombinase in mouse embryonic fibroblast reporter cell line, expressed by functional activation of GFP at 48 h post‐transfection. b) Selected polymers (highlighted in blue after exclusion of false positives) showed comparable transfection efficiency to Lipofectamine 2000. Non‐cytotoxic polymers inducing less Cre‐mediated recombination than naked Cre mRNA were excluded from this analysis. Data are expressed as mean ± SEM (n = 3–14). c) Chemical structures of common bisacrylamides and amines in identified hits. d) Transfection efficiency of NPs containing mRNA Cre recombinase after polymer purification by dialysis. (d.1) Cre‐mediated recombination was quantified by high‐content imaging. Data are expressed as mean ± SEM (n = 6–17). (d.2) Representative fluorescence microscope images of lead NP candidate (P1E28) compared to Lipofectamine 2000. Scale bars = 100 µm. Data in (b) and (d.1) were analyzed by one‐way ANOVA with post hoc Dunnett's multiple comparisons test against free mRNA and untreated controls, respectively: (*), *p* < 0.05; (**), *p* < 0.01; (***), *p* < 0.001; (****), *p* < 0.0001.

Our polymer library was synthesized via Michael‐type addition of a fixed hydrophobic diacrylate (P1) with chemically diverse bisacrylamides (A–E) and amines (1–32), dispersed in dimethyl sulfoxide (DMSO) at a molar ratio of 1:1:2, respectively. Chemical structures of the monomers used in this study are described in Figure S1, Supporting Information. Finally, these terpolymers were end‐capped with an excess 10% molar ratio of the respective amines, in order to improve polymer biocompatibility and transfection efficiency.^[^ ^13^, ^20^ ^]^ This synthetic approach enabled the rapid generation of 152 polymers out of 160 possible combinations for complexation with mRNA, with the remaining 8 combinations unable to be formed due to gelation. NP formulations were prepared in nuclease‐free water by mixing each newly synthesized polymer (dissolved in DMSO) with mRNA encoding Cre recombinase at a fixed mass ratio of 100:1, respectively. High‐throughput screening was performed in 96‐well plates by treating a mouse embryonic fibroblast reporter cell line with polymeric NPs (50 ng/well Cre mRNA) dispersed in serum‐free cell culture medium. Lipofectamine 2000 was used as a positive control following its demonstration of effective mRNA delivery in different cell models.^[^ ^21^ ^]^ Successful mRNA delivery to these cells resulted in the expression of Cre recombinase, which excises the *loxP*‐flanked STOP cassette and ultimately enables gene expression of GFP (**Figure** **1**a).^[^ ^22^ ^]^ High‐content imaging enabled simultaneous analysis of transfection efficiency and cytotoxicity (Figure S2, Supporting Information). Most formulations were well‐tolerated by the reporter fibroblasts, despite the presence of residual DMSO (< 1% v/v). Although this solvent has been employed in previous screening studies of polymeric NPs and showed negligible toxicity at the concentrations used herein,^[^ ^23^ ^]^ these polymers can also be prepared in aqueous solutions prior to complexation with mRNA,^[^ ^24^ ^]^ in order to facilitate their clinical translation. From the 52 non‐cytotoxic formulations with superior transfection efficiency than naked mRNA alone (Figure 1b), 2 polymers (P1C06, P1A05) induced comparable gene recombination to commercial Lipofectamine 2000, followed by 4 other hits (P1E28, P1E04, P1C05, P1A04) which also induced significant GFP expression compared to vector‐free mRNA treatment (*p* < 0.012). P1D21 (*p* = 0.0397) was excluded after visual inspection of microscopy images because the quantified green fluorescence was due to polymer autofluorescence rather than GFP expression after transfecting with Cre mRNA (Figure S2c, Supporting Information). Hence, we selected 6 polymers that shared chemical features such as the incorporation of linear alkyl amines 4, 5, or 6, differing primarily by the combination with bisacrylamides A, C, or E (Figure 1c). These alkyl amines are readily protonated at physiological pH, thus conferring high cationic charge density, which may improve the electrostatic complexation of mRNA and NP uptake.^[^ ^2b^ ^]^ P1E28 strikingly contrasted from the remaining polymers with the incorporation of an alkyl alcohol side chain combined with the presence of bisacrylamide E, which is characterized by piperazine rings containing two tertiary amines conferring the polymer with high buffering capacity.^[^ ^11^, ^25^ ^]^

Activity of the selected polymers was further validated after purification by dialysis in DMSO (MWCO = 2 kDa), in order to remove free monomers and short polymer chains that might compete for electrostatic complexation with mRNA and thus reduce the number of functional polyplexes. The mRNA:polymer mass ratio of the polymeric nanoformulations was adjusted accordingly to minimize cytotoxicity (Figure S3, Supporting Information). Interestingly, P1E28 polymer emerged as the lead candidate (Figure 1d.1), inducing superior cell recombination in fibroblasts (Figure 1d.2) than Lipofectamine 2000 (62.1 ± 3.3% vs 45.1 ± 10.4%), followed by P1A05 (39.0 ± 11.7%) and P1A04 (32.5 ± 6.2%) which induced comparable gene recombination. The performance of P1E28 was not sensitive to batch‐to‐batch variation (*p* = 0.4611, n = 4 batches, data not shown).

### 2.2 Hit Validation Against Several Human Cell Types Using Different mRNA Molecules

**Figure 2:**
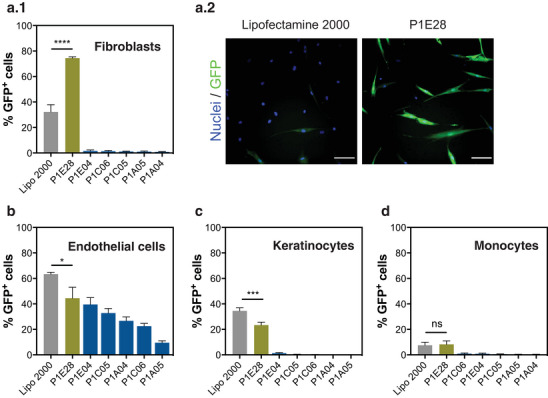
Hit validation and cellular tropism. a) Identified polymers were used to transfect GFP mRNA in human dermal fibroblasts. (a.1) Transfection efficiency and (a.2) representative microscope images of human dermal fibroblasts transfected with GFP mRNA. Scale bars = 100 µm. b) Transfection of GFP mRNA in human endothelial cells, c) human keratinocytes, and d) human monocytes. Data are expressed as mean ± SEM (n = 3) except for monocytes (n = 6). One‐way ANOVA with post hoc Tukey's multiple comparisons test was performed: (ns), *p* > 0.05; (*), *p* < 0.05; (***), *p* < 0.001; (****), *p* < 0.0001.

The hit formulations were then evaluated across several human cell types using a second mRNA, in this case encoding GFP protein. Because our mouse fibroblast reporter cell line is highly sensitive to the transfection of mRNA encoding Cre recombinase, GFP mRNA validated the polymer efficacy to deliver quantifiable amounts of mRNA functionally expressing protein, based on its fluorescence. P1E28 exhibited superior performance in inducing GFP expression than the remaining polymer candidates across the 4 tested cell types (**Figure** **2**), albeit only in human dermal fibroblasts (74.5 ± 1.6%) this formulation outperformed Lipofectamine 2000 (*p* < 0.0001). Notably, P1E28 transfected human dermal fibroblasts more efficiently than endothelial cells (44.5 ± 8.6%), keratinocytes (23.4 ± 2.2%), and monocytes (8.2 ± 2.7%), which suggested its preferential tropism towards fibroblasts. The remaining hits failed to achieve significant transfection efficiency levels across cell types except in endothelial cells.

**Figure 3:**
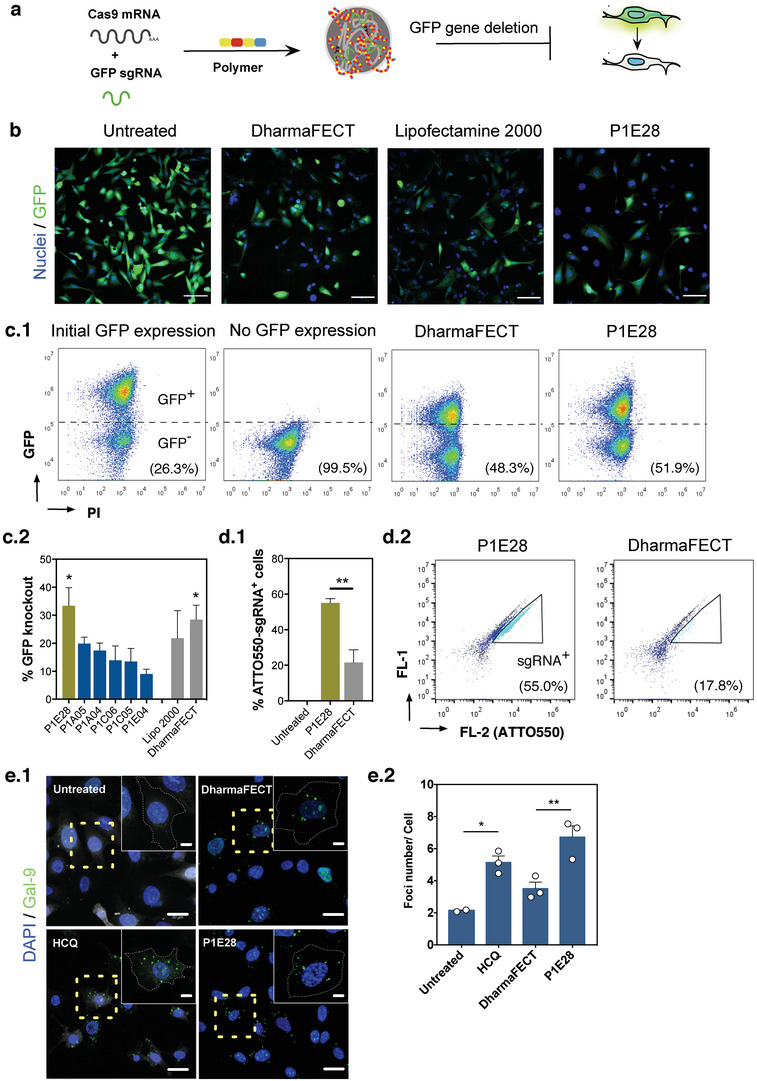
Development of an RNA‐based CRISPR/Cas9 delivery system. a) CRISPR/Cas9 is based on the delivery of Cas9 (as mRNA) alongside a guide RNA (sgRNA). Gene edition was performed on mouse embryonic fibroblast reporter cell line after Cre‐mediated recombination to promote stable GFP expression. Gene edition resulted in decreased GFP expression in transfected cells. b) Representative images of GFP knockout induced by P1E28 alongside respective controls, obtained at day 3 post‐transfection using high‐content imaging. Scale bars = 100 µm. c) Long‐term effects of gene edition quantified at day 10 post‐transfection using flow cytometry. (c.1) Representative flow cytometry scatter plots of cells treated with P1E28 formulation alongside controls demonstrate GFP knockout. (c.2) Percentage of GFP knockout normalized to the initial GFP expression, assessed by flow cytometry. GFP knockout was calculated after normalizing the number of GFP negative cells in each treatment compared to the untreated control. Data are expressed as mean ± SEM (n = 3–6). One‐way ANOVA with post hoc Dunnett's multiple comparisons test was performed: (*), *p* < 0.05. d) Cellular uptake of NPs complexed with Cas9 mRNA and a fluorescently labeled sgRNA (ATTO550) by dermal fibroblasts was assessed by flow cytometry 4 h after transfection. (d.1) Percentage of cells internalizing NPs with ATTO550‐labelled sgRNA and (d.2) representative scatter plots of cells treated with P1E28 or DharmaFECT Duo. Data are expressed as mean ± SEM (n = 3). One‐way ANOVA with post hoc Tukey's multiple comparisons test was performed: (**), *p* < 0.01. e) Endosomal disruption induced by P1E28 was measured by quantifying the formation of galectin‐9 foci. (e.1) Representative confocal microscopy images of reporter fibroblasts 4 h after incubation with P1E28 or DharmaFECT Duo. Hydroxychloroquine (HCQ) was used as a positive control (60 µm). Scale bars = 20 µm (insets = 6.5 µm). (e.2) Number of galectin‐9 foci per cell. Data are expressed as mean ± SEM (n = 2–3 independent experiments, with 3–4 confocal images acquired for each replicate). One‐way ANOVA with post hoc Tukey's multiple comparisons test was performed: (*), *p* < 0.05; (**), *p* < 0.01.

We further investigated our validated hits in the context of gene editing. In addition to Cre recombinase, the CRISPR/Cas9 system has emerged as a promising tool for precise genome modifications, as it uses tunable sgRNAs to direct the enzyme to any target site in genomic DNA.^[^ ^3a^ ^]^ Hence, CRISPR/Cas9 systems based on RNA delivery require the delivery of both mRNA and sgRNA, which have significant differences in size (molecular weight) and charge density (**Figure** **3**a). Because mRNA encoding Cas9 is much longer (4 kb) than Cre mRNA and GFP mRNA (approximately 1 kb), this required further optimization of the delivery system, namely the doses of vector, mRNA, and sgRNA (Figure S4, Supporting Information). In order to compare the transfection performance of gene editing tools (CRISPR/Cas9 and Cre recombinase) in the same cell type, we transfected the reporter fibroblast cell model with Cre recombinase protein to stably express GFP in approximately 80% of the treated cells. Functional delivery of Cas9 mRNA and sgRNA targeting GFP was evaluated by high‐content imaging, 72 h after transfection (Figure 3b), in order to assess the knockout of GFP fluorescence after gene editing by non‐homologous end joining repair upon DNA excision. As positive controls, we have used 3 different commercial agents (Lipofectamine 2000, Lipofectamine RNAiMAX, DharmaFECT Duo), with variable capacities to load nucleic acids with different sizes, to deliver both Cas9 mRNA and sgRNA (Figure S4b, Supporting Information). Lipofectamine 2000 and DharmaFECT Duo were superior than Lipofectamine RNAiMAX at the optimal formulation using mRNA:sgRNA ratio = 1:1. Polymer candidates achieved optimal transfection using 4 times less sgRNA than lipid‐based vectors to complex with the same dose of mRNA, resulting in a ratio mRNA:sgRNA = 1:0.25 (Figure S4b,c, Supporting Information). Furthermore, separate delivery of mRNA and sgRNA using different vectors did not offer any benefit to the overall transfection (Figure S4d, Supporting Information), hence we proceeded with the complexation of both nucleic acids in the same vector to ensure they both reach the same target cell.^[^ ^7b^ ^]^

Long‐term efficacy of CRISPR/Cas9 gene edition was evaluated by flow cytometry at day 10 post‐transfection, in order to validate initial results demonstrating decreased fluorescence after excision of GFP induced by Cas9 (Figure 3c.1).^[^ ^26^ ^]^ All formulations were shown to have a positive effect in the reduction of GFP fluorescence, indicating that genomic edition of GFP was performed. Nevertheless, only P1E28 (33.4 ± 6.5%, *p* = 0.0112) and DharmaFECT Duo (28.4 ± 5.1%, *p* = 0.0407) significantly increased the abundance of GFP‐negative cells compared to the untreated control (Figure 3c.2). In line with our previous findings using mRNA encoding Cre recombinase and GFP, P1E28 emerged as the top performing candidate for co‐delivery of Cas9 mRNA and sgRNA. This formulation was more efficient than DharmaFECT Duo because it required 4 times less sgRNA for the same mRNA dose to achieve comparable GFP knockout. This could be explained by the superior cellular uptake of P1E28 polyplexes. When formulated with the same amounts of Cas9 mRNA and a labeled GFP sgRNA, cells treated with P1E28 exhibited an average fluorescence intensity 3.4 times higher (*p* = 0.0145) compared to DharmaFECT Duo (Figure S5, Supporting Information), suggesting efficient polyplex uptake by dermal fibroblasts. These findings were further demonstrated by flow cytometry (Figure 3d), showing that 55.0 ± 2.4% of the cells effectively internalized P1E28, compared to only 21.5 ± 7.2% with DharmaFECT Duo (*p* = 0.0099).

Effective mRNA delivery requires not only that the nanoformulation is taken up by cells but also escapes the endosomal compartment and releases its cargo to the cytosol. We performed intracellular trafficking studies to further determine how P1E28 compares with commercial transfection agents. To determine the capacity of P1E28 to escape the endosomal compartment we monitored the induction of galectin‐9 foci after NP internalization (Figure 3e). This protein has been reported to sense endosomal vesicle disruption.^[^ ^27^ ^]^ Cells treated with P1E28 showed an increased number of galectin‐9 foci compared to those treated with DharmaFECT Duo (*p* = 0.0090). These results were comparable to the treatment with hydroxychloroquine (HCQ), which was used as a positive control (*p* = 0.0221 vs negative control), suggesting that P1E28 could rapidly escape from the endosomal compartment within 4 h after transfection.

All polymers in our library incorporate an *o*‐nitrobenzyl‐based diacrylate (P1) in the polymer backbone, which renders it sensitive to UV radiation. Considering its effective degradation and subsequent release of short RNAs after UV irradiation,^[^ ^13^, ^16^ ^]^ we evaluated the responsiveness of P1E28 formulated with Cre mRNA to UV radiation (*λ* = 365 nm, 10 mW cm^−2^, 10 min). Although we clearly identified polymer cleavage and degradation upon UV exposure, this did not result in enhanced transfection efficiency (Figure S6, Supporting Information). On the contrary, UV radiation hindered mRNA release, which could be attributed to covalent crosslinking between the generated aldehydes after polymer degradation and terminal alcohols in the polymer's side chains and in the ribose backbone of the complexed mRNA (Figure S7, Supporting Information).

### 2.3 In Vivo Validation of Lead Polymeric Formulation During Acute Wound Healing in the Skin

**Figure 4:**
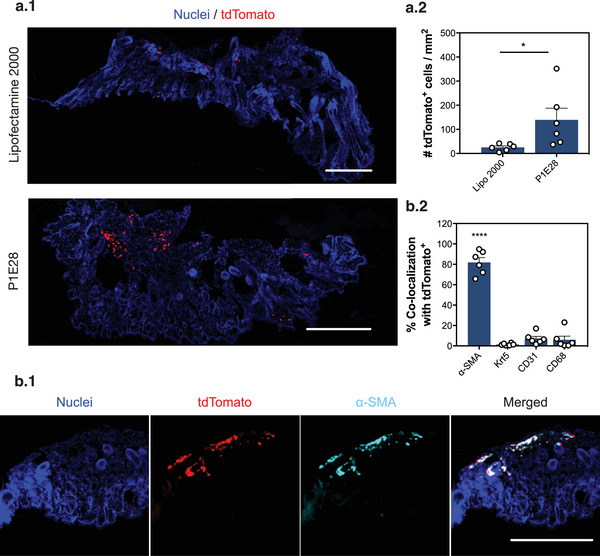
In vivo validation of targeted mRNA delivery to skin fibroblasts mediated by P1E28 during wound healing. a) R26‐tdTomato mice were intradermally injected with Cre mRNA delivered by P1E28 or Lipofectamine 2000. Skin samples were obtained from wounds at day 7 post‐injection. (a.1) Representative fluorescence microscope images of skin sections illustrate the transfection efficiency of P1E28, generating tdTomato^+^ cells (red) after Cre‐mediated recombination. Scale bars = 500 µm. (a.2) Quantification of generated tdTomato^+^ cells normalized by tissue section area. Data are expressed as mean ± SEM (n = 6 animals, 2 wounds per animal). Unpaired two‐sample t‐test was performed: (*), *p* < 0.05. b) Tropism of P1E28 polyplexes was characterized by immunohistochemical staining of skin sections containing tdTomato+ cells. (b.1) Representative fluorescence microscope images illustrate that the vast majority of *α*‐SMA+ activated fibroblasts (light blue) co‐localized with tdTomato fluorescent areas (red). Scale bars = 500 µm. (b.2) Co‐localization of each cell type with tdTomato was obtained after calculating the Manders’ overlap coefficient. Data are expressed as mean ± SEM (n = 6 animals, 3–5 images per animal). One‐way ANOVA with post hoc Tukey's multiple comparisons test was performed: (****), *p* < 0.0001.

We validated in vivo the improved performance of P1E28 in the delivery of Cre mRNA compared to lipid‐based transfection agents, in the context of acute wound healing (**Figure** **4**). To test our hypothesis that P1E28 polyplexes would preferentially accumulate in skin fibroblasts (Figure 2), these polymeric NPs complexed with Cre mRNA were injected intradermally around the wound (4 different locations) in R26‐tdTomato mice at day 5 post wounding, when the granulation tissue is established to restore blood supply and to promote regeneration of the skin epithelium.^[^ ^28^ ^]^ Similarly to SFr cells used in our screening approach (Figure 1a), this model contains a *loxP*‐flanked STOP cassette which can be excised by Cre recombinase, resulting in the expression of fluorescent tdTomato (Figure 4a.1). Skin sections were harvested 7 days post injection to allow for the Cre‐mediated recombination process to occur. The administered nanoformulations had no impact in physiological wound closure (Figure S8, Supporting Information). P1E28 elicited the recombination of approximately 5.5 times more tdTomato fluorescent cells (139.2 ± 48.9 cells mm^−2^ vs 25.3 ± 9.5 cells mm^−2^, *p* = 0.0419) than Lipofectamine 2000 (Figure 4a.2). These regions confirming effective mRNA delivery were further analyzed by immunohistochemistry to characterize cell targets of P1E28 polyplexes (Figure 4b.1 and Figure S9, Supporting Information). The tdTomato signal evidencing areas of Cre‐mediated recombination was strongly co‐localized with activated fibroblasts (82.0 ± 4.8%), which typically express *α*‐smooth muscle actin (*α*‐SMA) in acute skin wounds (Figure 4b.2).^[^ ^17^ ^]^ In contrast, endothelial cells (CD31^+^, 7.4 ± 2.6%), macrophages (CD68^+^, 5.9 ± 3.4%), and keratinocytes (Krt5^+^, 1.6 ± 0.7%) were poorly co‐localized with tdTomato, supporting the preferential tropism of P1E28 towards fibroblasts (p < 0.0001) as observed in vitro.

### 2.4 Structure‐Activity Relationships Predict Efficacy of mRNA Delivery

**Figure 5:**
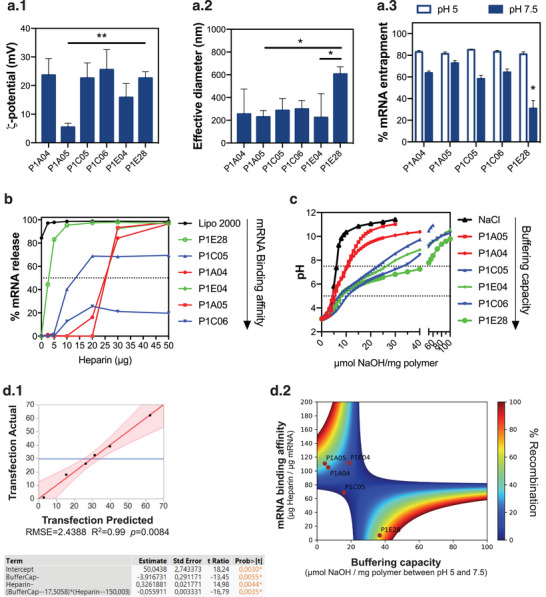
Structure‐activity relationships of polymeric NPs for mRNA delivery. a) Physicochemical properties of the identified hits: (a.1) surface charge, (a.2) hydrodynamic diameter, and (a.3) complexation efficiency. Results are expressed as mean ± SEM (n = 3). In (a.1) and (a.2), data were analyzed by one‐way ANOVA with post hoc Tukey's multiple comparisons test: (*), *p* < 0.05; (**), *p* < 0.01. In (a.3), data were analyzed by two‐way ANOVA with post hoc Sidak's multiple comparisons test: (*), *p* < 0.05. b) Heparin replacement assay estimated the polymers’ binding affinity to Cre mRNA after quantification of fluorescence intensity of bands in each lane corresponding to a different heparin dose. Data represent the mean value of 2–3 gel replicates. Horizontal dashed line corresponds to 50% mRNA release. c) Acid‐base titration for the selected polymers dissolved in 150 mm NaCl, adjusted for pH 3 with HCl. d) Multiple linear regression of polymer buffering capacity and mRNA binding affinity enabled the correlation of these parameters with transfection efficiency. (d.1) Standard least squares effect leverage with two‐way interactions described the significant interaction of these parameters in transfection efficiency, with negative coefficient Heparin*Buffer indicating an inversely proportional relationship. (d.2) Heatmap representation of the predicted model with highlighted experimental points corresponding to selected polymers.

In order to understand the differences among the identified hits with respect to their cellular activity, we characterized polymeric NPs prepared by electrostatic complexation with Cre mRNA (**Figure** **5**). All formulations rendered positively charged NPs, with *ζ*‐potential values ranging between +5 and +25 mV (Figure 5a.1) and average particle sizes between 200 and 600 nm (Figure 5a.2). All polymers except P1A05 revealed a significantly greater complexation efficiency at pH 5 compared to pH 7.5 (*p* < 0.0017), which suggested the predominance of electrostatic interactions ensuring complexation efficiencies around 60% at physiologically relevant pH 7.5 (Figure 5a.3). This could be attributed to the presence of primary amines both in the side chains and end‐caps.^[^ ^29^ ^]^ Interestingly, despite its high transfection efficiency, the complexation efficiency of P1E28 was the lowest among the selected polymers, and decreased from 80.9 ± 2.2% at pH 5 to 30.8 ± 7.4% at pH 7.5. Nevertheless, all polyplexes maintained their colloidal stability in nuclease‐free water for at least 96 h, and in serum‐free medium used to transfect the reporter fibroblasts for 4 h (Figure S10, Supporting Information). Supplementation of cell culture medium with serum proteins (FBS) did not significantly affect the colloidal stability of NP formulations.

The affinity of mRNA to the polymeric formulations was measured by a heparin replacement assay, which consists of the destabilization of electrostatic interactions in the presence of competing negative charges (Figure 5b). P1E28 and Lipofectamine 2000 presented extremely low binding affinity towards mRNA, suggesting that this polymer could readily release its cargo. Binding affinity and particle size were not significantly affected by the mRNA cargo (Figure S11, Supporting Information), despite differences between Cre recombinase and GFP mRNA in length (1350 vs 996 nucleotides) and GC content in the coding sequence (68.8% vs 61.5%), which influence the formation of secondary structures and thus may affect how mRNA is complexed with these polymers.^[^ ^30^ ^]^ Nonetheless, the selected polymers seemed to have lower complexation efficiency of GFP mRNA compared to Cre mRNA (Figure S11, Supporting Information), even though this was only statistically significant for P1C05 (*p* = 0.0004). Notably, P1E28 had the lowest complexation efficiency among the identified hits.

Endosomal escape of polymeric NPs is highly correlated with the polymer buffering capacity to overcome vesicle acidification.^[^ ^11d^ ^]^ Acid‐base titration showed that the selected polymers are capable of proton buffering in physiological pH (Figure 5c), with estimated pKa values between 4.1 and 4.8 (Figure S11, Supporting Information). Among all polymers, P1E28 was the one that buffered the most protons between pH 5 and pH 7.5, corresponding to a buffering capacity of 36.8 µmol H^+^ mg^−1^ polymer. We hypothesized that the remarkably low binding affinity of mRNA to P1E28 and the polymer's high buffering capacity determined its efficacy towards mRNA intracellular delivery, considering that we did not observe any correlation between biological activity (i.e., Cre‐mediated recombination) and these factors for the remaining polymers (Figure S12, Supporting Information). Similarly, polymer physicochemical properties such as particle number, size, or surface charge were poor predictors of biological activity.

We reasoned that NP activity could not be predicted by a sole factor, but rather by the combination of these parameters. Hence, we performed multiple linear regression using a standard least squares model to assess whether mRNA binding affinity and polymer buffering capacity played a role in NP activity (Figure S13, Supporting Information). We obtained a statistically significant model (*p* = 0.0084) describing that both parameters negatively affected each other and determined the formulation's performance (Figure 5d.1 and Figure S13, Supporting Information). The design space generated from our experimental results consisted of two major categories (Figure 5d.2): i) polymers with low buffering capacity (< 20 µmol NaOH mg^−1^ polymer) and high mRNA binding affinity (> 100 µg heparin µg^−1^ mRNA); or ii) polymers with high buffering capacity (> 30 µmol NaOH mg^−1^ polymer) and low mRNA binding affinity (< 100 µg heparin µg^−1^ mRNA). The first category corresponds to polymers constituted by alkyl amines, which confer a greater cationic content that enables the uptake of greater amounts of complexed mRNA (Figure 5a.3). However, their low buffering capacity may preclude NP release from acidified endosomes, leading to their degradation. Here, the selected cationic polymers performed best in endothelial cells (Figure 2b), where they could exploit alternative endocytic mechanisms such as caveolae‐mediated pathways,^[^ ^31^ ^]^ which target instead the Golgi and the endoplasmic reticulum.^[^ ^32^ ^]^ Further investigation on the influence of endocytic pathways in mRNA delivery to each cell type is warranted to maximize transfection efficiency. Another aspect that merits further consideration is the fact that a high binding affinity may be detrimental for mRNA release to the cytosol. In order to circumvent that issue, cationic polymers can be designed with shorter chains to weaken their electrostatic interactions with mRNA.^[^ ^33^ ^]^

In contrast with the remaining polymers, P1E28 fits in the latter category representing an ideal candidate to overcome some of the major challenges in polymer‐based vectors: i) entrapment in the endolysosomal compartment, ii) dissociation and release of mRNA to the cytosol. While its low binding affinity was comparable to commercially available transfection agents (Figure 5b) and is associated with efficient mRNA release for protein translation,^[^ ^21^, ^34^ ^]^ P1E28 may benefit from its high buffering capacity to promote endosomal escape to the cytosol. The capacity of P1E28 to destabilize the endosomal compartment (Figure 3e) can also be explained by its strong ionization at endosomal pH,^[^ ^30b^ ^]^ as suggested by the sharp difference in mRNA complexation between pH 7.4 and pH 5 (Figure 5a.3). These properties seemed to be dictated by the unique combination of the 3 different monomers constituting the polymer (Figure S14, Supporting Information), as transfection efficiency was abrogated when the hydrophobic diacrylate (P1) was removed and the bisacrylamide moiety (E) replaced by the other molecules used to synthesize the library. Interestingly, the length of the alkyl chain of the amino alcohol moiety (28) also determined polymer performance, with longer chains favoring transfection. While hydrophobicity may be an important feature determining polymer stability, we hypothesized that these chemical features could also determine the apparent selectivity of P1E28 toward fibroblasts (Figure 3d).

### 2.5 Proposed Molecular Mechanism Behind the NP Targeting to Fibroblasts

**Figure 6:**
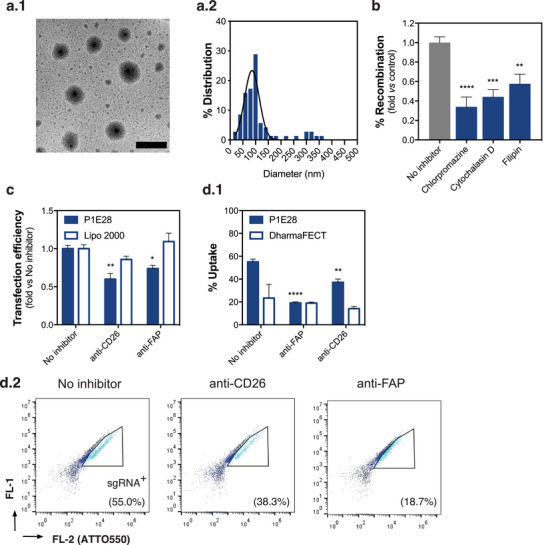
Molecular mechanism of P1E28 formulation for fibroblast targeting. a) Size of polyplexes generated by complexation of P1E28 with Cre mRNA. (a.1) Representative TEM image and (a.2) NP diameter distribution show the formation of small NPs (87% of measured NPs < 200 nm). Scale bar = 500 nm. b) Recombination in mouse reporter fibroblast cell model after endocytosis inhibition of P1E28 via clathrin‐ (chlorpromazine) and caveolin‐mediated endocytosis (filipin III), as well as actin‐related processes (cytochalasin D). Data are expressed as mean ± SEM (n = 5–6). One‐way ANOVA with post hoc Dunnett's multiple comparisons test was performed: (**), *p* < 0.01; (***), *p* < 0.001; (****), *p* < 0.0001. c) Transfection efficiency was determined by quantifying the number of cells expressing GFP, after delivery of GFP mRNA to human dermal fibroblasts with or without receptor blocking using monoclonal antibodies targeting CD26 and FAP. Data are expressed as mean ± SEM (n = 3). Two‐way ANOVA with post hoc Sidak's multiple comparisons test was performed: (*), *p* < 0.05; (**), *p* < 0.01. d) Impact of receptor blocking on cellular uptake of NPs complexed with Cas9 mRNA and a fluorescently labeled sgRNA (ATTO550) was assessed by flow cytometry. (d.1) Percentage of cells internalizing NPs with ATTO550‐labelled sgRNA 4 h after transfection and (d.2) representative scatter plots of cells treated with P1E28 with or without antibody blocking. Data are expressed as mean ± SEM (n = 3). Two‐way ANOVA with post hoc Sidak's multiple comparisons test was performed: (**), *p* < 0.01; (****), *p* < 0.0001.

P1E28 polyplexes were characterized with respect to their size and potential mechanisms of internalization (**Figure** **6**). Gel permeation chromatography showed that P1E28 polymer was characterized by a narrow polydispersity and molecular weight around 5 kDa (Mn = 4949, Mw = 6167, Mw/Mn = 1.246). In contrast to their large hydrodynamic diameter in water and in cell culture medium (Figure 5a.2 and Figure S10, Supporting Information), transmission electron microscopy (TEM) images (Figure 6a.1) showed that most of the NPs formulated by complexation of P1E28 with mRNA had a diameter up to 200 nm (Figure 6a.2). Pre‐treatment of fibroblasts with chlorpromazine, cytochalasin D and filipin inhibited Cre‐mediated recombination by about 66%, 56%, and 42%, respectively, after the delivery of mRNA encoding Cre recombinase (Figure 6b). These results indicated that the activity of P1E28 NP formulations required the involvement of different endocytic mechanisms, including clathrin‐ and caveolae‐dependent pathways,^[^ ^35^ ^]^ as well as actin‐dependent processes which are not only involved in uptake (e.g., macropinocytosis) but also in intracellular trafficking.^[^ ^36^ ^]^

Considering the greater relevance of clathrin‐dependent pathways, we reasoned that the enhanced activity of P1E28 in fibroblasts was attributed to receptor‐mediated endocytosis. In the context of wound healing, *α*‐SMA^+^ activated fibroblasts derive from reticular fibroblasts overexpressing CD26,^[^ ^17^ ^]^ which are associated with fibrotic processes involving the secretion of extracellular matrix and constitute approximately 28% of the fibroblasts populating the human dermal skin.^[^ ^37^ ^]^ However, while CD26 is enriched in these fibroblasts during wound healing, it is also expressed by pro‐regenerative papillary fibroblasts and keratinocytes.^[^ ^37^, ^38^ ^]^ A recent study showed that CD26^+^ reticular fibroblasts also presented high levels of FAP, whose expression is restricted to reactive fibroblasts in the skin.^[^ ^39^ ^]^ Considering that CD26 and FAP share roughly 50% of sequence homology,^[^ ^40^ ^]^ we first validated their expression in the fibroblast cell models used in this work (Figure S15, Supporting Information). While CD26 was expressed in both the mouse embryonic fibroblasts (SFr reporter fibroblasts) and human dermal fibroblasts (AG08468), the expression of FAP was much higher in the latter. Both receptors can be pharmacologically targeted by small molecules used as catalytic inhibitors.^[^ ^41^ ^]^ P1E28 shares some chemical features with these molecules, including the presence of aromatic (promote *π*‐*π* stacking interactions with S1 pocket) and piperazine rings (mediate salt bridges with S2 pocket), in addition to the hydrogen bonds established by the polymer's carbonyl groups and amines.

Therefore, we interrogated whether CD26 and FAP were involved in the uptake of P1E28 polyplexes by blocking the receptors with their respective antibodies (Figure 6c,d). Blocking CD26 and FAP in human dermal fibroblasts significantly hampered the transfection efficiency of P1E28 by 40% (*p* = 0.0059) and 24% (*p* = 0.0337), respectively, whereas the isotype control did not have a significant impact in transfection (*p* = 0.1531). Similarly, Lipofectamine 2000 was not affected by receptor blocking (p = 0.6243). These results were in agreement with the impaired uptake of P1E28 polyplexes after blocking CD26 (*p* = 0.0066) and FAP (*p* < 0.0001), whereas lipid‐based DharmaFECT Duo was not affected (*p* = 0.3845). Because cellular uptake of polymeric NPs may decrease in the presence of serum,^[^ ^34^ ^]^ we evaluated the influence of serum proteins (FBS) in particle uptake and gene editing activity of polymeric NPs prepared by complexation of P1E28 with Cas9 mRNA and sgRNA (Figure S16, Supporting Information). Although the presence of serum resulted in more negatively charged NPs, thus reducing NP uptake, it did not significantly affect the gene editing activity of P1E28 (*p* = 0.2474). Collectively, these results suggest that the polymeric NPs formulated by complexation of P1E28 with mRNA offer a promising solution for specific in vivo mRNA delivery to profibrotic fibroblasts by targeting CD26 and FAP receptors.

## 3 Conclusion

In the present study, we report the development of a polymeric NP‐based formulation for intracellular delivery of mRNA with tropism for fibroblasts. The implication of CD26 and FAP in NP uptake pose an attractive target for mRNA delivery to fibroblasts involved in fibrotic disorders in the skin, liver, lung, and heart,^[^ ^39^, ^42^ ^]^ as well as in autoimmune diseases such as rheumatoid arthritis and Crohn's disease.^[^ ^43^ ^]^ This formulation constitutes an alternative to NP functionalization with anti‐FAP antibodies, which have been employed for the modulation of the microenvironment of epithelial tumors.^[^ ^44^ ^]^ To the best of our knowledge, this is the first time a nanoformulation is described with tropism for fibroblasts for efficient mRNA delivery without the incorporation of specific targeting ligands. These findings may inspire the development of future formulations targeting specific cell receptors, based on the incorporation of monomers mimicking small molecule inhibitors. Although LNPs, such as the ones used for mRNA vaccines, have shown remarkable efficiency in vivo, they offer less options in terms of chemical and physical diversity than the current polymeric formulations for cell targeting.

Importantly, the identified polymers shared some chemical features resulting either in high cationic content for efficient mRNA complexation or in high buffering capacity to enable endosomal escape and mRNA release to the cytosol. Further investigation using other reporter cell types could expand this high‐throughput screening methodology to identify novel formulations with specific cell tropism, and to validate the impact of polymer chemistry strategies (e.g., branching, end‐capping) on NP uptake and effective mRNA delivery to specific cell types.^[^ ^11^, ^45^ ^]^

## 4 Experimental Section

### Synthesis of Polymer Library

Monomers used for polymer synthesis were purchased from different suppliers as described in Table S1, Supporting Information. Polymers were synthesized via Michael addition reaction, as previously reported.^[^ ^13a^ ^]^ Briefly, all monomers were diluted to 1.6 m in anhydrous dimethyl sulfoxide (DMSO, Alfa Aesar), prior to the combination of the different bisacrylamides (50 µL of A‐E) and amines (100 µL of 1–32) with 50 µL of diacrylate linker (P1). Reaction mixtures were added to a polypropylene 96‐deep well plate (VWR), sealed with aluminum foil, and kept at 60 °C for 5 days in an incubator with an orbital shaker at 250 rpm. The resulting polymers were then capped with 20 µL of the respective amine (1–32) for 3 h under the same conditions, corresponding to 10% molar excess. Polymer libraries were stored at −20 °C until further use. All polymers were described by their constituent monomers (e.g., polymer constituted by diacrylate P1, bisacrylamide A, and amine 1 is abbreviated to P1A01, which is end capped by the same amine 1).

### Nanoparticle Size and Charge

Polyplex size and *ζ*‐potential were measured by dynamic light scattering (DLS) using a ZetaPALS Analyzer (Brookhaven Instruments Corporation). Polyplexes were prepared by mixing for 10 min the respective polymer with 1 µg Cre mRNA in milliQ ultrapure water at the optimized mass ratios, followed by dilution in ultrapure water to a final volume of 1 mL. All size measurements were performed using the same incident laser power and recorded with a scattering angle of 90°, following an equilibration time of 5 min. Each measurement corresponded to the average result of 5 runs of 1 min each. An aliquot of 100 µL of each sample was collected for nanoparticle tracking analysis (NTA). After restoring samples with the same volume of ultrapure water, *ζ*‐potential measurements were performed by maximizing incident laser power for each sample with at least 5 runs presenting a relative residual value below 0.05. NTA was performed using a Nanosight LM10 system (Malvern Panalytical) after diluting aliquoted samples to 1 mL in ultrapure water. Samples were loaded into the chamber using a syringe pump at 10 µL min^−1^. Five videos of 1 min each were recorded for each sample. Camera level was set at 16 and detection threshold at 4. Data analysis was performed using the NTA software (version 2.2, Malvern Panalytical).

### Polymer Acid‐Base Titration

Buffering capacities of polymers were determined after dissolving 1 mg polymer in 5 mL 0.1 m sodium chloride (Fisher Scientific) and adjusting the solutions to pH 3 using 1 m hydrochloric acid (Fisher Scientific). Titration of a 5 mL sample of 0.1 m sodium chloride lacking polymer was performed as a background control. The solution pH was measured after each addition of 0.01 m sodium hydroxide (Merck) using an inoLab 720 pH meter (WTW GmbH). Buffering capacity was calculated by normalizing the amount of NaOH buffered between pH 5 and 7.5 to the total polymer mass. The derivative of the titration curves was obtained in order to determine the effective pKa, which corresponds to the pH at which the normalized buffering capacity was the highest.^[^ ^29^ ^]^

### Gel Permeation Chromatography

Gel permeation chromatography was performed using a size exclusion chromatography (SEC) setup from Viscotek (Viscotek TDAmax) equipped with differential viscometer (DV), right‐angle laser‐light scattering (RALLS, Viscotek), and refractive index (RI) detectors. The column set consisted of a Viscotek Tguard column (8 µm) followed by one Viscotek T4000 column, one Viscotek D2000 column, and one Waters column WAT044232. A dual piston pump was set at a flow rate of 1 mL min^−1^. The eluent (dimethylformamide with 0.03% LiBr) was previously filtered through a 0.2 µm filter. The system was also equipped with an on‐line degasser. The tests were done at 60 °C using an Elder CH‐150 heater. Before the injection (100 µL), the samples were filtered through a polytetrafluoroethylene membrane with 0.2 µm pore. The system was calibrated with narrow polymethylmethacrylate standards (20–9680 Da). Molecular weight (Mn SEC) and dispersity (Ð = Mw/Mn) of the synthesized polymers were determined by TriSEC calibration using the OmniSEC software (Malvern Panalytical, version 4.6.1.354).

### TEM Imaging of P1E28 Nanoparticles

Purified P1E28 polymer (0.5 mg mL^−1^) was complexed with Cre, GFP, and Cas9 mRNAs (with GFP sgRNA at 1:0.25 mRNA:sgRNA mass ratio) at a 1:20 mRNA:polymer mass ratio in molecular‐grade, nuclease‐free water. A droplet of each sample was deposited on carbon‐coated grids and left in a closed Petri dish at room temperature to dry for 5 h. TEM was performed using a JEOL‐2100‐HT electron microscope, equipped with a fast‐readout “OneView” 4k x 4k CCD camera operating at 25fps (300 fps with 512×512 pixel) and featuring drift correction. NP size distribution was carried out by manual counting on ImageJ software on several TEM images.

### RNA Complexation Efficiency

Polyplexes were prepared by incubating polymers with 100 ng/well mRNA (encoding Cre recombinase or GFP) in nuclease‐free water for 10 min, as described above. Polyplexes were then diluted to a volume of 100 µL/well in a buffer comprised of 150 mm sodium chloride (Fisher Scientific), 20 mm sodium phosphate (Sigma), 20 mm sodium acetate (Merck), and 25 mm sodium citrate (Sigma), adjusted at pH 5 or pH 7.5, as previously described.^[^ ^30b^ ^]^ Samples were prepared in triplicate at the respective pH. Standards were prepared by diluting the respective mRNA to doses ranging from 0.25 to 1 µg mL^−1^ in the buffer set at the respective pH. Following the manufacturer's instructions, SYBR Gold (Invitrogen) was diluted 1:100 prior to the addition of 1 µL per 100 µL polyplex (final dilution 1:10 000). Yellow fluorescence resulting from the hybridization of SYBR Gold to free mRNA was measured in opaque black well‐plates, using a Synergy H1 plate reader (Biotek) set with excitation wavelength = 495 nm and emission wavelength = 537 nm. Entrapment of mRNA was calculated by subtracting the amount of mRNA used to generate polyplexes (100 ng) by the amount of detected free mRNA (based on the linear calibration curve obtained from the standards).

### Heparin Replacement Assay

Purified polymer candidates were complexed with 250 ng Cre mRNA at the optimized mRNA:polymer mass ratios for 10 min. Polyplexes were then incubated for 30 min at 37 °C with 2.5–50 µg of heparin (Calbiochem), corresponding to heparin:mRNA ratio ranging from 10:1–200:1. Polyplex destabilization in the presence of negatively charged heparin resulted in the release of mRNA, which was measured by agarose gel electrophoresis. Free mRNA was used as a control for electrophoretic migration upon polyplex dissociation. Agarose gels were prepared by dissolving 1% (w/v) agarose (ultrapure grade, NZYTech) in TBE 1x and staining the molten gel with 1:10 000 SYBR Gold (Invitrogen) to visualize mRNA bands. Samples were loaded with 5 µL RNA loading dye 2x (Thermo Scientific), totaling a loading volume of 25 µL in each well. Electrophoresis was performed at 100 V for 30 min. Gels were imaged immediately after completing electrophoresis using a ChemiDoc XRS system (BioRad) equipped with a UV light transilluminator. Complexation studies were repeated 2–3 times for each sample.

### UV‐Triggered Polyplex Dissociation and mRNA Release

Light sensitivity of P1E28 was investigated after preparing 1 mL samples containing 20 µg polymer and 1 µg Cre mRNA in milliQ ultrapure water, as described above. Samples were irradiated using a UV lamp (*λ* = 365 nm, 100 W) for 10 min, at an incident power density of 10 mW cm^−2^. Original and irradiated samples were measured by DLS and NTA. Aliquots of 50 µL were extracted to monitor UV‐triggered polymer degradation by the generation of nitrosobenzene derivatives by UV/Vis spectrophotometry, using a Synergy H1 plate reader (Biotek). Effect of UV irradiation in polymer degradation was investigated by diluting P1E28 in deuterated DMSO (DMSO‐d_6_, Sigma Aldrich) followed by UV irradiation as described above. UV‐triggered polyplex dissociation was performed in deuterated water (D_2_O, Merck Millipore), followed by dilution in DMSO‐d_6_. Samples were analyzed by ^1^H nuclear magnetic resonance (NMR) using a Bruker Avance III spectrometer (400 MHz). Residual non‐deuterated DMSO was used as an internal reference for ^1^H chemical shifts (ppm). Polyplex stability was evaluated by heparin replacement assay as described above. Briefly, samples were irradiated for 10 min at 10 mW cm^−2^, prior to the addition of different doses of heparin for 30 min at 37 °C. Polyplex dissociation was measured by agarose gel electrophoresis.

### Cell Culture

Polyplex screening was performed using SFr cells derived from SC‐1 mouse fibroblasts stably transduced with a retroviral SF91‐loxP1‐RFP‐loxP2‐eGFP construct.^[^ ^22^ ^]^ These cells were kindly offered by Dr. Carol Stocking (University of Hamburg, Germany). SFr cells were cultured in DMEM medium (Corning) supplemented with 10% (v/v) fetal bovine serum (FBS, Life Technologies) and 0.5% (v/v) penicillin/streptomycin (VWR). Cellular tropism was validated using human dermal fibroblasts (AG08468, Coriell Institute for Medical Research; passages: 17–22) and human keratinocytes (HaCaT, passages: 32–35), both cultured in supplemented DMEM, as well as human monocytes (THP‐1, passages: 7–11) cultured in supplemented RPMI 1640 (Gibco), and human umbilical vein endothelial cells (HUVECs, passages: 9–13) cultured in supplemented EGM‐2 medium (Lonza). Cells were grown at 37 °C, 5% CO_2_ to 80–90% confluency, and passaged every 3–4 days. Transfections were performed 24 h after seeding cells in each experiment.

### High‐Throughput Screening

SFr cells (2500 cells/well) were seeded on a 96‐well plate (Costar) 24 h prior to mRNA transfection. Polyplexes were prepared in sterile 96‐deep well polypropylene plates (VWR) by diluting each polymer in DMSO and mixing with 50 ng/well mRNA encoding Cre recombinase, modified with 5‐methoxyuridine (TriLink Biotechnologies, cat. no. L‐7211), dissolved in sterile‐filtered, nuclease‐free water (Life Technologies) to a total volume of 10 µL/well. After incubation under agitation for 10 min, the generated polyplexes were diluted with 90 µL/well of serum‐free DMEM for transfection. An initial optimization was performed using a selected polymer (P1C05) from a previous study,^[^ ^13a^ ^]^ a mRNA:polymer mass ratio of 1:100 (i.e., 1 µL polymer in DMSO at 5 mg mL^−1^) was chosen for the selected dose of 50 ng/well mRNA (0.5 µg mL^−1^), resulting in a polymer dose of 5 µg/well (50 µg mL^−1^) for the library screening. As a positive control, 0.25 µL/well Lipofectamine 2000 (Invitrogen) was complexed with the same mRNA dose, according to the manufacturer's instructions (volume:mass ratio = 5 µL Lipofectamine µg^−1^ mRNA). After 4 h of incubation, cells were washed to remove any remaining particles in the media and replenished with complete DMEM. After culturing for 48 h, cells were stained with Hoeschst H33342 and propidium iodide (PI), both at 1 µg mL^−1^ (Sigma) in DMEM, and analyzed in a high‐content microscope (InCell Analyzer 2200, GE Healthcare). Cellular recombination was assessed by determining the number of cells expressing GFP, using an excitation filter of 475 nm (28 nm aperture) and an emission filter of 511.5 nm (23 nm aperture).

### Hit Validation

Identified polymer candidates were purified by dialysis in DMSO (Fisher Scientific) using pre‐wetted regenerated cellulose membranes with MWCO = 2 kDa (Spectrum Spectra/Por 6 Standard RC Dialysis Tubing, Fisher Scientific). Mass concentrations of purified polymers were determined after further dialysis in milliQ ultrapure water (Merck Millipore) and lyophilization by freeze‐drying. SFr cells were treated with purified polymers (5–300 µg mL^−1^) for 4 h, followed by PI staining after 48 h to evaluate polymer cytotoxicity using the InCell Analyzer 2200 microscope. Transfection of mRNA encoding EGFP, modified with 5‐methoxyuridine (TriLink Biotechnologies, cat. no. L‐7201), was performed under the aforementioned conditions for Cre mRNA. Different GFP mRNA:polymer ratios were tested in SFr cells using non‐cytotoxic polymer concentrations. Mouse SFr fibroblasts (2500 cells/well), human AG08468 dermal fibroblasts (3000 cells/well), HaCaT keratinocytes (3000 cells/well), and HUVEC endothelial cells (3000 cells/well) were seeded 24 h prior to transfection. THP‐1 monocytes (10 000 cells/well) were activated with 100 ng mL^−1^ phorbol 12‐myristate 13‐acetate (Sigma Aldrich) for 72 h in order to adhere to the 96‐well plate, followed by 24 h of rest by washing the culture medium prior to transfection. Transfection efficiency was evaluated in mouse SFr fibroblasts, human AG08468 dermal fibroblasts, HaCaT keratinocytes, HUVEC endothelial cells, and THP‐1 macrophages, by quantifying the number of cells expressing GFP in a similar fashion to the aforementioned analysis of Cre‐mediated recombination, using the InCell Analyzer 2200 microscope.

### Delivery of Cas9 mRNA and sgRNA

SFr‐GFP cells were generated in a 24‐well plate after transfection of SFr cells (40 000 cells/well, passage 13) with 500 ng Cre recombinase (New England Biolabs) complexed with 1.5 µL Lipofectamine RNAiMAX (Life Technologies) in serum‐free DMEM for 24 h, followed by another 24 h of culture in complete medium.^[^ ^46^ ^]^ The resulting SFr‐GFP cells (recombination efficiency = 80%) were further expanded for subsequent experiments. SFr‐GFP cells were seeded in 96‐well plates (3000 cells/well) 24 h prior to transfection. Polymers were first evaluated in the co‐delivery of Cas9 mRNA, modified with 5‐methoxyuridine (TriLink Biotechnologies, cat. no. L‐7206), and a synthetic single guide RNA targeting GFP, with targeting sequence 5’‐GGCCACAAGUUCAGCGUGUC‐3’ (CRISPRevolution sgRNA EZ kit, Synthego). Polyplex stability was evaluated at different mRNA:polymer and mRNA:sgRNA ratios by agarose gel electrophoresis. Co‐delivery of mRNA and sgRNA was optimized using 2 different mRNA doses (50–200 ng/well) and 4 mRNA:sgRNA ratios (1:0.25–1:2) and 3 different commercial transfection agents (volume:mass ratio = 5 µL µg^−1^ mRNA), according to the respective manufacturers’ instructions: Lipofectamine 2000, Lipofectamine RNAiMAX and DharmaFECT Duo (Dharmacon, GE Healthcare). A fixed mRNA dose of 100 ng/well (1 µg mL^−1^) was selected for similar mRNA:sgRNA optimization using polymers. Finally, mRNA:polymer mass ratios were optimized with a fixed mRNA dose of 100 ng/well and mRNA:sgRNA = 1:0.25. Co‐delivery of mRNA and sgRNA was compared with sequential delivery of the same mRNA dose and optimized mRNA:polymer ratio, followed by the delivery of 25 ng/well of sgRNA using Lipofectamine 2000, 6 or 24 h after mRNA transfection. GFP knockdown was quantified by high‐content imaging 72 h after the first transfection, using the InCell Analyzer 2200 microscope. SFr‐GFP cells were then expanded to 6‐well plates and kept for 10 days after co‐delivery of mRNA and sgRNA. Sustained GFP knockdown was measured by flow cytometry. Cells were dissociated with 0.05% (w/v) trypsin‐EDTA in PBS 1x (Gibco) and centrifuged at 300 g for 3 min. Cell pellets were re‐suspended in 250 µL PBS 1x and analyzed using a BD Accuri C6 flow cytometer (BD Biosciences), equipped with 488 and 640 nm lasers. Dead cells labeled with PI staining and GFP expression of live cells were quantified using the 675/25 and 530/30 filters, respectively. Data were processed using FlowJo software (version X.0.7, FlowJo LLC).

### Cellular Uptake

Internalization of P1E28 complexed with Cas9 mRNA and GFP sgRNA was compared to the commercial agent DharmaFECT Duo (Dharmacon, GE Healthcare) using an ATTO550‐labelled sgRNA, derived from the hybridization of an ATTO550‐labelled tracrRNA and a crRNA targeting GFP (both from Integrated DNA Technologies). Briefly, both RNA molecules were mixed to a final concentration of 30 µm and incubated for 5 min at 95 °C, followed by 20 min at room temperature, in order to allow hybridization of the labeled sgRNA. Particle uptake was evaluated by high‐content imaging and flow cytometry.

For high‐content imaging, human dermal fibroblasts (AG08468) were seeded in a µ‐Slide 8‐well (IBIDI) chambered coverslip (9375 cells/well). In order to compare polyplex internalization by fluorescence intensity, both P1E28 and DharmaFECT Duo were complexed with the same doses of Cas9 mRNA and sgRNA (150 ng/well mRNA and 37.5 ng/well; mRNA:sgRNA ratio 1:0.25). P1E28 (mRNA:polymer mass ratio = 1:20; 3 µg/well) or DharmaFECT Duo (5 µL µg^−1^ mRNA; 0.75 µL/well) were incubated in the RNA mix for 10 min prior to transfection in DMEM with or without FBS for 4 h, as described above. After this time, cells were washed 3 times to remove non‐internalized polyplexes and stained with DAPI (1 µg mL^−1^; Sigma Aldrich) in phosphate buffered saline (PBS) 1x for 5 min. ATTO550 fluorescence was evaluated using the InCell Analyzer 2200 microscope.

For flow cytometry, human dermal fibroblasts (AG08468) were seeded in 12‐well plates (30 000 cells/well). After 24 h, both P1E28 and DharmaFECT Duo were complexed with a fixed dose of Cas9 mRNA (300 ng/well) and ATTO550‐labelled sgRNA (75 ng/well, mRNA:sgRNA ratio = 1:0.25). Briefly, P1E28 (mRNA:polymer mass ratio = 1:20; 6 µg/well) or DharmaFECT Duo (5 µL µg^−1^ mRNA; 1.5 µL/well) were incubated in the RNA mix for 10 min prior to transfection in serum‐free DMEM for 4 h, as described above. Cells were then washed with a heparin solution (50 µg mL^−1^) in PBS 1x, before dissociation with 0.05% (w/v) trypsin containing ethylenediaminetetracetic acid (EDTA) in PBS 1x (Gibco) and centrifugation at 300 g for 3 min. Cell pellets were re‐suspended in 250 µL PBS 1x and analyzed using a BD Accuri C6 flow cytometer (BD Biosciences). Uptake of ATTO550 was quantified using the 585/40 and 530/30 filters to compensate for cell autofluorescence. Data were processed using FlowJo software (version X.0.7, FlowJo LLC).

### High‐Content Analysis

Cell viability and GFP expression were quantified by live cell imaging using the InCell Analyzer 2200 microscope. Seven fields per well were acquired with a 20x objective using the laser autofocus method on the following channels: DAPI/Hoeschst 33 342 (exposure: 0.6 s, excitation/emission: 390/435 nm), FITC/GFP (exposure: 0.4 s, excitation/emission: 475/511 nm), Texas Red/PI (exposure: 0.2 s, excitation/emission: 575/620 nm). TIFF images produced for each channel were processed using the InCell Investigator software (GE Healthcare). Cells were identified using the top‐hat method in the DAPI channel (minimum area = 80 µm^2^, sensitivity = 93), followed by classification of viability according to the cell nuclei. Dead cells were identified by nuclear condensation (Nuc Intensity CV < 0.4) and co‐localization with PI staining, determined by pseudo‐segmentation in the Texas Red channel. Collar segmentation with a set radius of 3 µm in the FITC channel to identify GFP fluorescent cells, presenting cytoplasmic (perinuclear) fluorescence intensity greater than the acquired background (Reference 1 Cell/Bckg Intensity > 1.03). Analysis parameters of high‐content imaging were adjusted for HUVEC and THP‐1 cells (sensitivity = 31, Nuc Intensity CV < 0.45, Cell/Bckg Intensity > 1.05).

Uptake of ATTO550‐labelled NPs by human dermal fibroblasts was assessed using the same software to perform a top‐hat selection in the DAPI channel (minimum area = 30 µm^2^, sensitivity = 70). ATTO550 fluorescent foci were quantified in an ROI delineated around the identified cell nuclei using a collar segmentation with a set radius of 5 µm, in order to account for the heterogeneous distribution of foci with different sizes and intensities. Results were expressed as the average intensity (arb. units) in the ROI and were normalized to untreated negative control.

### Endocytosis Inhibition

Lead polymer candidate P1E28 was complexed with Cre mRNA for transfection in SFr cells, as described above. Uptake inhibition was investigated by comparing transfection efficiency of this polyplex with cells pre‐treated for 1 h with inhibitors of clathrin‐mediated endocytosis (chlorpromazine hydrochloride, 10 µg mL^−1^; Sigma Aldrich), caveolae‐mediated endocytosis (filipin III, 3 µm; Sigma Aldrich), and actin‐mediated processes such as macropinocytosis (cytochalasin D, 10 µm; Sigma Aldrich). These conditions were selected based on relevant studies on the cellular uptake of polyplexes and were not cytotoxic to SFr cells (data not shown).^[^ ^32^, ^35^ ^]^ Polyplexes added to cells were prepared in DMEM containing the same concentration of inhibitors. Lipofectamine 2000 was used as a positive control in the absence or presence of these inhibitors. Live GFP fluorescent cells were quantified as described above, using the InCell Analyzer 2200 microscope.

### Receptor Blocking for Uptake and Transfection Studies

AG08468 human dermal fibroblasts were seeded in 96‐well plates (3000 cells/well) for transfection experiments or in 12‐well plates (30 000 cells/well) for uptake evaluation. Cells were pre‐treated for 1 h with rabbit anti‐human CD26 (1:200; cat. no. 67138S, Cell Signaling Technology) or with rabbit anti‐human FAP (1:200; cat. no. 66562S, Cell Signaling Technology) in serum‐free DMEM, in order to block the receptors. A rabbit IgG fraction was used as an isotype control (1:1000; cat. no. X0903, Dako) to account for non‐specific antibody interactions. P1E28 and Lipofectamine 2000 were complexed with GFP mRNA for transfection, as described above. For uptake experiments, the complexes were prepared using Cas9 mRNA and ATTO550‐labelled sgRNA, as described above. Particle uptake was assessed 4 h after transfection by flow cytometry. Transfection efficiency in the absence or presence of these blockers was compared 24 h after transfection by quantifying live GFP fluorescent cells, using the InCell Analyzer 2200 microscope.

### Immunocytochemical Analysis of Fibroblasts

To assess the expression of FAP and CD26, SFr mouse reporter fibroblasts and AG08468 human dermal fibroblasts were seeded in a µ‐Slide 8‐well (IBIDI) chambered coverslip (21 250 cells/well). Cells were gently rinsed with PBS 1×24 h after seeding and fixed with ice‐cold methanol for 15 min. Cells were washed thrice with PBS for 5 min each, and permeabilized with 0.3% Triton X‐100 in PBS for 10 min. After a new washing step (3×5 min with PBS), cells were incubated with blocking buffer (1% BSA and 0.3 m glycine in PBS 1x) for 1 h, followed by an overnight incubation at 4 °C with rabbit anti‐human CD26 (1:200; cat. no. 67138S, Cell Signaling Technology) or with rabbit anti‐human FAP (1:100; cat. no. 66562S, Cell Signaling Technology) diluted in PBS 1x containing 1% BSA and 0.3% Triton X‐100. After washing with PBS (3×5 min), cells were incubated in the dark at room temperature with a goat anti‐rabbit antibody labeled with AlexaFluor 488 (1:1000; cat. no. A11034; Invitrogen) diluted in PBS 1x containing 1% BSA for 1 h. After another washing step with PBS 1x (3×5 min), sections were stained with DAPI (1 µg mL^−1^; Sigma Aldrich) for 5 min and washed with PBS 1x before imaging.

To assess the disruption of endolysosomes, SFr mouse reporter fibroblasts were seeded in a µ‐Slide 8‐well (IBIDI) chambered coverslip (21 250 cells/well). Cells were treated with P1E28 or DharmaFECT Duo complexed with a fixed dose of Cas9 mRNA (100 ng/well) and ATTO550‐labelled sgRNA (25 ng/well) in serum‐free DMEM for 4 h. As a positive control, cells were treated with 60 µm HCQ under the same conditions. Cells were washed with a heparin solution (50 µg mL^−1^) in PBS 1×4 h after transfection and fixed with 4% paraformaldehyde for 10 min, followed by permeabilization with 0.3% Triton X‐100 and blocking with 1% BSA and 0.3 m glycine, as described above. Cells were incubated for 1 h with the rat anti‐galectin 9 (1:100; cat. no. 137 901, BioLegend) diluted in PBS 1x containing 1% BSA, followed by a washing step with PBS to remove the primary antibody. Cells were then incubated with the secondary chicken anti‐rat IgG Alexa Fluor 488 (1:1000; cat. no. A21470; Invitrogen) diluted in PBS 1x containing 1% BSA for 1 h, and the nuclei stained with DAPI (1 µg mL^−1^; Sigma Aldrich) for 5 min.

For both experiments, cells were imaged using a Zeiss LSM 710 confocal microscope, equipped with an Apochromat 40x/1.4 objective. Images were analyzed in ImageJ (version 1.51, NIH) to count individual galectin‐9 foci and measure fluorescence intensity levels of CD26 and FAP in each cell using the Analyze Particles built‐in function.

### Experimental Animals

Nineteen‐week‐old male and female (22–24 g) R26‐tdTomato reporter mice (C57BL/6 mice bearing a loxP‐flanked STOP cassette impeding tdTomato expression) were used in the present study, in accordance with the ARRIVE guidelines, and after ethical approval from the Ethics Committee of the Faculty of Medicine of the University of Coimbra (ORBEA_159_2017/0 505 2017) and the Portuguese National Authority for Animal Health (DGAV project reference: 01 5433_0421/000/000/2017). Animals were maintained in groups of 5 with free access to water and food, under a regular 12 h light/dark cycle at a temperature of 19–22°C and relative humidity of 45–65%.

### Wound Induction

Animals were separated in individual cages 24 h before the induction of skin wounds. After anesthetizing by inhalation of 3% isoflurane in oxygen flowing at 2 L min^−1^, the dorsal skin of each mouse was shaved and disinfected using iodopovidone (Betadine). Two full‐thickness excision wounds (6 mm diameter) were inflicted 5 cm apart using a sterile biopsy punch in each animal. In order to minimize distress, analgesia (buprenorphine, 0.1 mg kg^−1^) was administered subcutaneously 30 min before wound induction, and every 6–8 h up to 48 h post wounding.

### In Vivo mRNA Delivery

At day 5 post wounding, P1E28 or Lipofectamine 2000 (both n = 6 mice; 3 male + 3 female) were complexed with 7 µg Cre mRNA (0.3 mg kg^−1^ mouse) for 10 min, prior to dilution in serum‐free DMEM to a final volume of 120 µL/mouse. Each treatment was administered by intradermal injection across 4 different locations around the wound (30 µL/injection). Two male mice (vehicle controls) were administered with the same volume containing only serum‐free DMEM. All animals were monitored daily for 12 days after wound induction. Wound healing was monitored by measuring the wound area over time. Mice were sacrificed 7 days post injection by cervical dislocation and skin samples were harvested for immunohistochemical analysis.

### Immunohistochemical Analysis of Skin Wounds

Skin samples were dissected with an excess area around the respective wound and fixed in 10% neutral buffered formalin overnight at 4 °C, with the subcutaneous tissue interfacing a small piece of cardboard to keep the sample flat. Fixed skin samples were embedded in optical cutting temperature (OCT) and snap‐frozen for cryo‐sectioning. Longitudinal sections were obtained using a Leica CM3050S cryotome set at a thickness of 10 µm, and stored at −80 °C until further processing. Skin sections were gently rinsed with PBS 1x to remove excess OCT, and permeabilized with 0.3% Triton X‐100 in PBS for 10 min. Sections were washed thrice with PBS for 5 min each, followed by a 1 h incubation in blocking buffer (5% BSA and 0.3 m glycine in PBS 1x) to block non‐specific interactions with proteins and unreacted aldehydes derived from fixation. After removing blocking solution, sections were incubated overnight at 4 °C with primary antibodies diluted in PBS 1x containing 1% BSA. After washing with PBS 1x (3×5 min), sections were incubated in the dark at room temperature with the respective secondary antibody diluted in PBS 1x containing 1% BSA for 1 h. After another washing step with PBS 1x (3×5 min), sections were stained with DAPI (1 µg mL^−1^; Sigma Aldrich) for 5 min and blot dried before mounting with fluorescence mounting medium (Dako). Sections were imaged using the InCell Analyzer 2200 microscope, set with a 20x objective. Images from entire sections were acquired and stitched together using the InCell Developer software (GE Healthcare). Skin samples obtained from the vehicle control were imaged in order to establish threshold levels for tissue autofluorescence. Cre‐mediated recombination was quantified in all wounds (n = 14) using ImageJ (version 1.51, NIH) to count individual cells with fluorescence intensity levels above the aforementioned thresholds. Fibroblasts were stained using rabbit anti‐*α*‐SMA (1:100; cat. no. ab5694; Abcam), followed by anti‐rabbit labeled with Cy3 (1:250; cat. no. 111‐165‐144; Jackson ImmunoResearch Europe Ltd). Endothelial cells were detected using goat anti‐CD31 (1:40; cat. no. AF3628; R&D Systems) followed by donkey anti‐goat labeled with AlexaFluor 488 (1:1000; cat. no. A11055; Invitrogen). Keratinocytes were identified using rabbit anti‐keratin 14 (1:1000; cat. no. 905 301; BioLegend) or chicken anti‐keratin 5 (1:200; cat. no. 905 901; BioLegend), followed by goat anti‐rabbit labeled with AlexaFluor 488 (1:1000; cat. no. A11034; Invitrogen) or anti‐chicken labeled with AlexaFluor 488 (1:1000; cat. no. A11039; Invitrogen), respectively. Macrophages were labeled using rat anti‐CD68 (1:100; cat. no. MCA1957; BioRad) followed by anti‐rat labeled with PE (1:500; cat. no. F0105B; R&D Systems). Co‐localization with the identified cell types was quantified in independent skin samples exhibiting Cre‐mediated recombination (i.e., tdTomato fluorescence) using the JACoP plugin on ImageJ, after adjusting fluorescence intensity levels to minimize autofluorescence artifacts. Co‐localization of each cell marker with the tdTomato signal was expressed as the Manders’ overlap coefficient x 100%.

### Statistical Analysis

All experiments were analyzed using GraphPad Prism software (version 7, GraphPad Inc.). Data were analyzed using 1‐ or 2‐way ANOVA tests with post hoc multiple comparisons test, provided statistical significance was obtained when *p* < 0.05. Multivariate analysis of polymer characteristics on its transfection efficiency was performed using JMP software (version 13, SAS). The data were fitted to a standard least squares model, set to affect leverage considering first‐order effects.

## Conflict of Interest

The authors declare the following competing interests: AFR and LF are applicants of a provisional Portuguese patent no. 117455. The remaining authors declare no conflict of interest.

## Author Contributions

A.F.R. and C.R. contributed equally to this work. A.F.R. and C.R. implemented the experiments and analyzed the data, under the supervision of L.F. and V.F. C.P. performed physicochemical characterization of polyplexes prepared using different mRNA molecules. S.S. performed immunofluorescence analysis. V.F. conceived the polymer library and characterized polymers by NMR and GPC. A.F.R., C.R., V.F., and L.F. conceived the overall design of the study. A.F.R. and L.F. wrote the manuscript, which was revised by all authors.

## Supporting information

Supporting Information

## Data Availability

The data that support the findings of this study are available from the corresponding author upon reasonable request.
