# Supplementary material for: A Polymeric Nanoparticle Formulation for Targeted mRNA Delivery to Fibroblasts
Source: Adv Sci (Weinh). 2022 Dec 18;10(5):2205475. doi: 10.1002/advs.202205475 (PMC9929262; doi:10.1002/advs.202205475)
Supplement: Supplementary file 1 — Supporting Information [file ADVS-10-2205475-s001.pdf]

A polymeric nanoparticle formulation for targeted mRNA delivery to fibroblasts

Artur Filipe Rodrigues[1,*], Catarina Rebelo[1,2,*], Susana Simões[1], Cristiana Paulo[1], Sónia Pinho[1], Vítor Francisco[1,#], Lino Ferreira[1,2,#]

[1] CNC – Center for Neurosciences and Cell Biology, University of Coimbra, 3000-517 Coimbra, Portugal

[2] Faculty of Medicine, Pólo das Ciências da Saúde, Unidade Central, University of Coimbra, 3000-354 Coimbra, Portugal

[*] These authors contributed equally to this study

[#] To whom correspondence should be addressed: francisco.vms@cnc.uc.pt ; lino@uc-biotech.pt

Supporting Information

Table S1. List of monomers used to synthesize polymer library.

Monomer	Name	CAS	Supplier
A	N,N'-Methylenebis(acrylamide)	110-26-9	Sigma Aldrich
B	1,6-Hexamethylenebis(methacrylamide)	16069-15-1	Sigma Aldrich
C	N,N'-Cystaminebis(acrylamide)	60984-57-8	Polysciences
D	N,N'-(1,2-Dihydroxyethylene)bis(acrylamide)	868-63-3	Sigma Aldrich
E	1,4-Bis(acryloyl)piperazine	6342-17-2	Sigma Aldrich
P1			In-lab, synthesized [13a]
1	Ethylenediamine	107-15-3	Merck
2	1,4-Diaminobutane	110-60-1	Sigma Aldrich
3	1,6-Diaminohexane	124-09-4	Alfa Aesar
4	Diethylenetriamine	111-40-0	Alfa Aesar
5	Triethylenetetraamine	112-24-3	Acros Organics
6	Pentaethylenehexamine	4067-16-7	Sigma Aldrich
7	3,3´-Diamino-N-methyldipropylamine	105-83-9	Sigma Aldrich
8	1,2-Diaminocyclohexane	694-83-7	Sigma Aldrich
9	1,8-Diamino-3,6-dioxaoctane	929-59-9	Acros Organics
10	1,13-Diamino-4,7,10-trioxatridecane	4246-51-9	Sigma Aldrich
11	1,4-Bis(3-aminopropyl)piperazine	7209-38-3	Sigma Aldrich
12	1,4-Bis(aminomethyl)benzene	539-48-0	Merck
13	1,5-Diaminonaphthalene	2243-62-1	Sigma Aldrich
14	4,4′-Diaminodiphenylmethane	101-77-9	Sigma Aldrich
15	1,3-Phenylenediamine	108-45-2	TCI Chemicals
16	1,3-Diaminopropane	109-76-2	TCI Chemicals
17	2,2-Dimethyl-1,3-propanediamine	7328-91-8	TCI Chemicals
18	1,3-Diaminopentane	589-37-7	TCI Chemicals
19	2,2'-Diamino-N-methyldiethylamine	4097-88-5	TCI Chemicals
20	Agmatine sulfate	2482-00-0	TCI Chemicals
21	1,4-Bis(aminomethyl)cyclohexane	2579-20-6	TCI Chemicals
22	4,4′-Methylenebis(cyclohexylamine)	1761-71-3	Sigma Aldrich
23	4,4`-Diaminobenzanilide	785-30-8	Sigma Aldrich
24	DL-Lysine monohydrochloride	70-53-1	Sigma Aldrich
25	3-Amino-1-propanol	156-87-6	Sigma Aldrich
26	4-Amino-1-butanol	13325-10-5	Sigma Aldrich
27	5-Amino-1-pentanol	2508-29-4	Sigma Aldrich
28	6-Amino-1-hexanol	4048-33-3	Alfa Aesar
29	1-(3-Aminopropyl)pyrrolidine	23159-07-1	Alfa Aesar
30	1-(3-Aminopropyl)imidazole	5036-48-6	Sigma Aldrich
31	1-(3-Aminopropyl)-4-methylpiperazine	224-954-4	Alfa Aesar
32	Histamine dihydrochloride	56-92-8	Sigma Aldrich

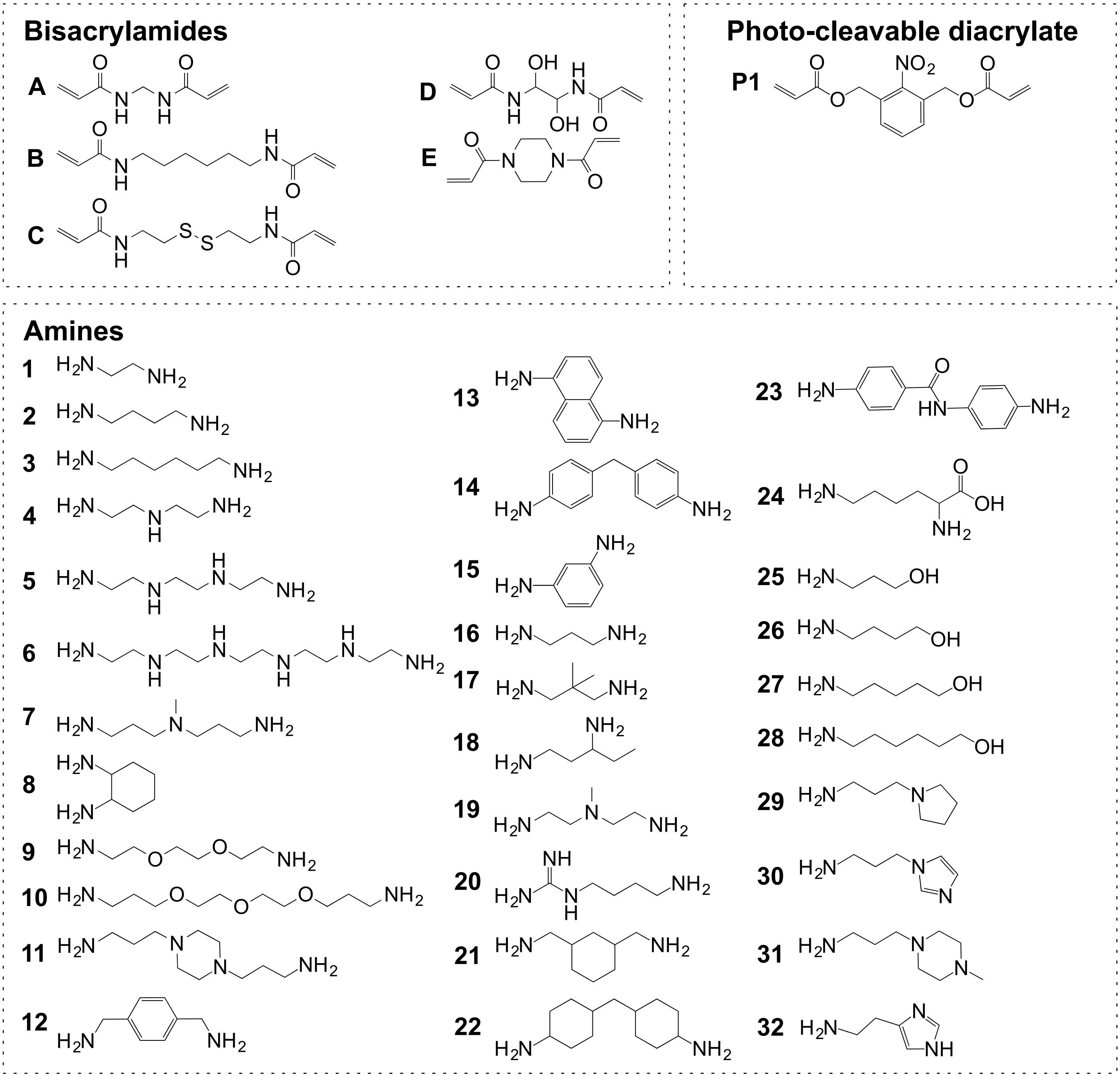

Figure S1. Schematic representation of monomers used to synthesize polymer library. Polymers were generated after combining P1 diacrylate with bisacrylamides (A-E) and amines (1-32).

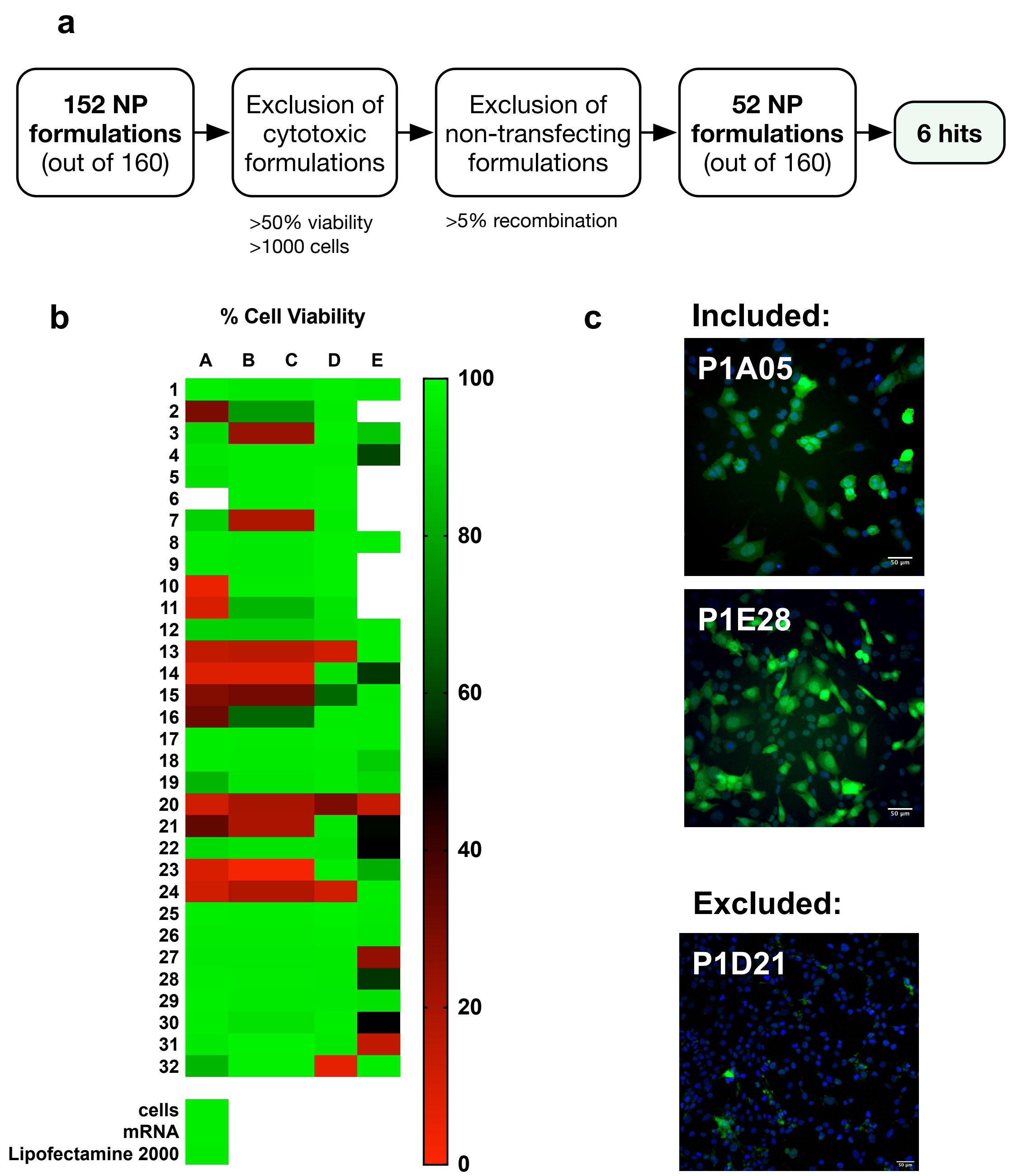

Figure S2. High-throughput screening of polymer library for mRNA delivery. (a) Screening strategy of the polymer library for the delivery of mRNA encoding Cre recombinase. **(b)** Heatmap representation of fibroblast cytotoxicity (based on nuclear condensation) of polymeric NPs illustrates the impact of the amine monomer, whereas the bisacrylamide had no effect on cell viability. **(c)** Transfection efficiency resulting in the expression of GFP upon Cre-mediated recombination was validated after careful analysis of microscopy images, which showed some false positives. Scale bars = 50 μm. The best true positives were highlighted with blue bars in **Figure 1b**.

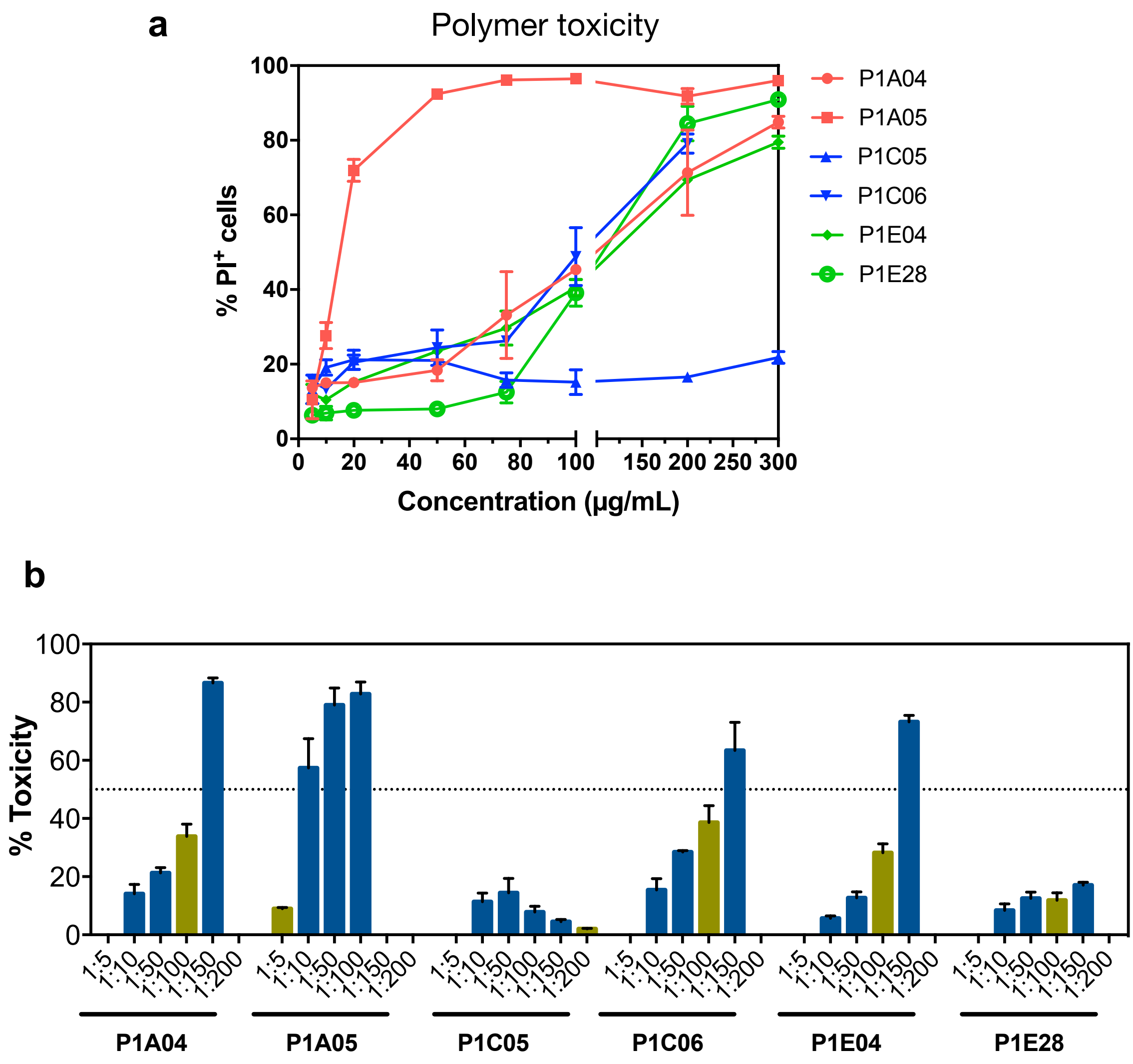

Figure S3. Cytotoxicity of top-performing polymers and NP formulations in fibroblasts. The polymers were purified by dialysis in DMSO using regenerated cellulose membranes (MWCO = 2 kDa). **(a)** Fibroblasts were incubated with the purified polymers alone at different doses to establish toxicological dose-response relationships by PI staining. **(b)** Toxicological dose-response curves of polymeric NPs (formulated by complexation with a fixed dose of 50 ng mRNA encoding GFP) supported polyplex optimization using different mRNA:polymer mass ratios. Selected ratios were highlighted in dark yellow, considering a cytotoxicity threshold at 50%, similarly as **Figure S2**. Data in **(a)** and **(b)** are expressed as mean ± SEM (n = 3).

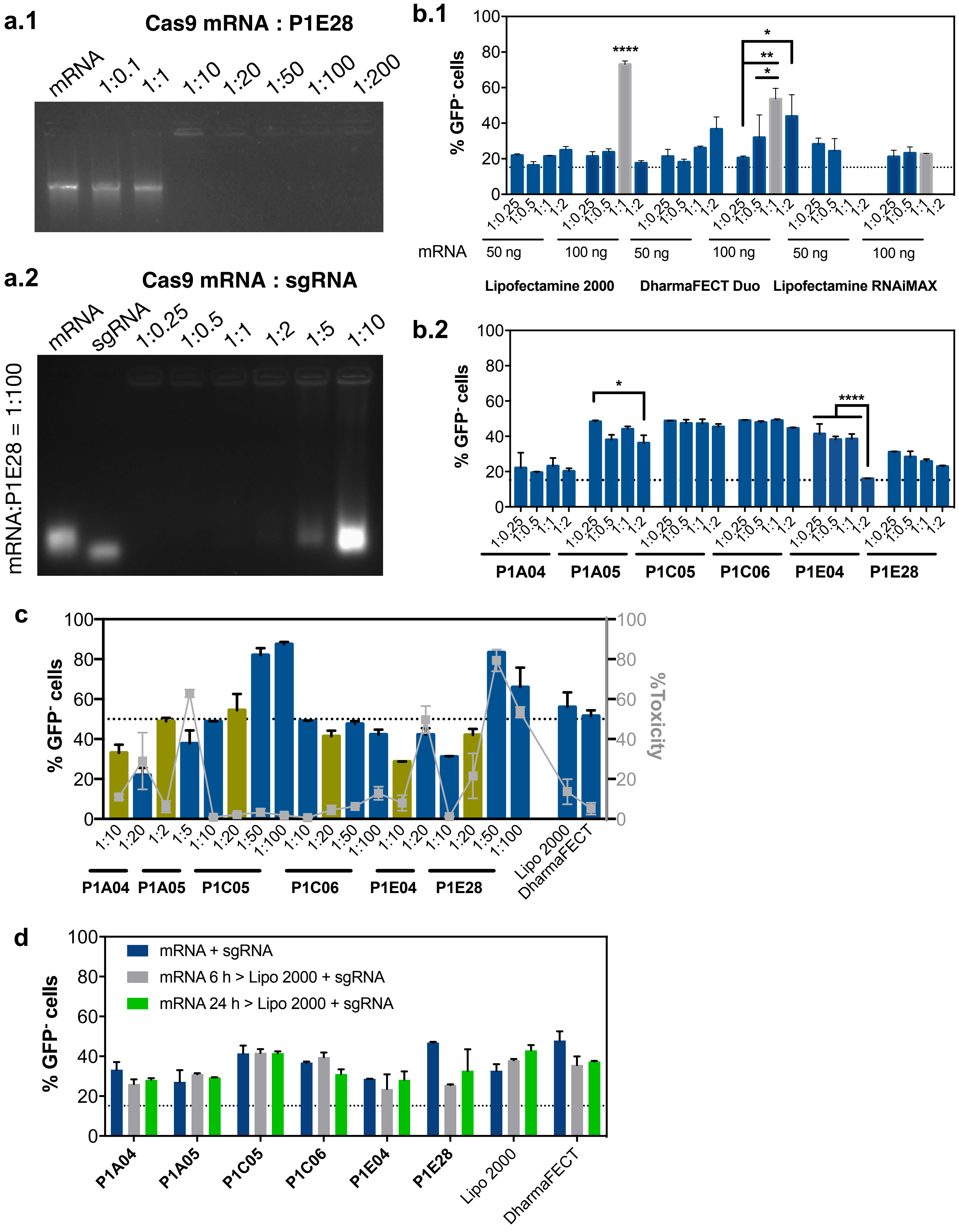

Figure S4. Optimization of polyplexes for co-delivery of Cas9 mRNA and GFP sgRNA. **(a)** Agarose gel electrophoresis of P1E28 complexed with (a.1) Cas9 mRNA and (a.2) GFP sgRNA determined optimal mass ratios to yield stable complexes. **(b)** Optimization of Cas9 mRNA and GFP sgRNA doses for co-delivery using (b.1) commercially available transfection agents and (b.2) the lead polymer candidates. First, mRNA and sgRNA doses were adjusted using the commercial agents by testing different mRNA:sgRNA mass ratios from 1:0.25 to 1:2. The selected doses for commercial agents were 100 ng mRNA + 100 ng sgRNA (mRNA:sgRNA ratio = 1:1). In the case of the tested polymers, the optimal sgRNA dose was 25 ng for 100 ng mRNA (mRNA:sgRNA = 1:0.25), indicating a greater efficiency than lipid-based agents in the delivery of RNA molecules for gene editing. Polymers were complexed at mRNA:polymer mass ratio of 1:10, except P1A05 due to its toxicity (1:2). Two-way ANOVA was performed with *post hoc* Tukey's multiple comparisons test: (*), $p < 0.05$; (**), $p < 0.01$; (****), $p < 0.0001$. **(c)** Optimization of polymer dose regarding transfection efficiency and cytotoxicity, using a fixed mRNA dose of 100 ng and mRNA:sgRNA ratio = 1:0.25. Selected mRNA:polymer ratios are highlighted in dark yellow, after exclusion of cytotoxic ratios (considering threshold at 50% in dashed line) and excessive polymer doses resulting in erroneous identification of GFP-negative cells due to autofluorescence in the DAPI channel (e.g. P1C05). **(d)** Comparison of co-delivery of Cas9 mRNA (100 ng) and sgRNA (25 ng) with staged delivery using Lipofectamine® 2000 to transfect sgRNA. Dashed line corresponds to the abundance of GFP-negative cells in the untreated control. No significant differences among delivery strategies were detected after performing two-way ANOVA with *post hoc* Tukey's multiple comparisons test. Data in **(b)**, **(c)**, and **(d)** are expressed as mean ± SEM (n = 2-4).

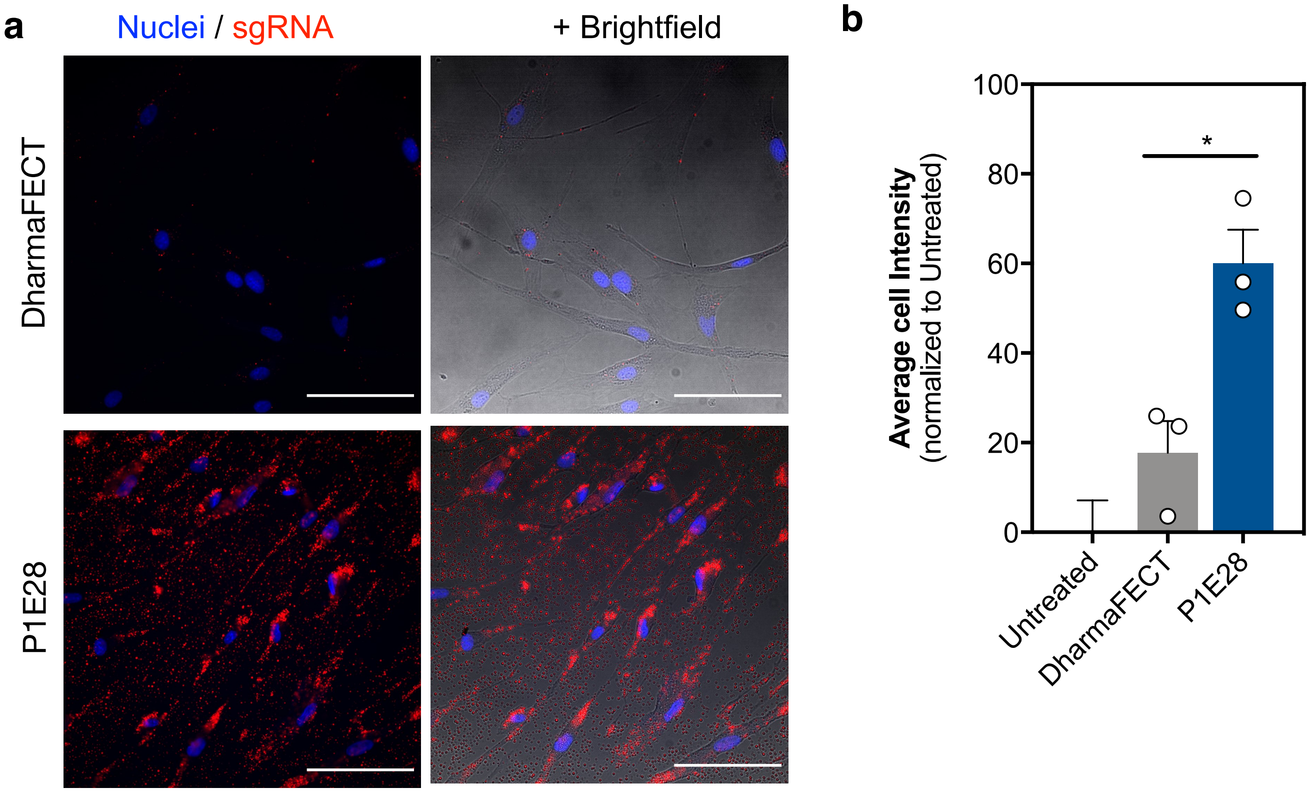

Figure S5. Cellular uptake of P1E28 complexed with Cas9 mRNA. Internalization of P1E28 complexed with Cas9 mRNA was compared to the commercial agent DharmaFECT Duo using an ATTO550-labelled sgRNA. **(a)** Representative fluorescence images show an increased ATTO550 signal in human dermal fibroblasts treated with P1E28 compared to DharmaFECT Duo. Scale bars = 50 µm. **(b)** Total fluorescence intensity of ATTO550 was normalized by the number of cells in each field. One-way ANOVA with *post hoc* Tukey multiple comparisons test was performed: (*), $p < 0.05$. Data are expressed as mean ± SEM (n = 3 independent measurements, each corresponding to the average of 7 images).

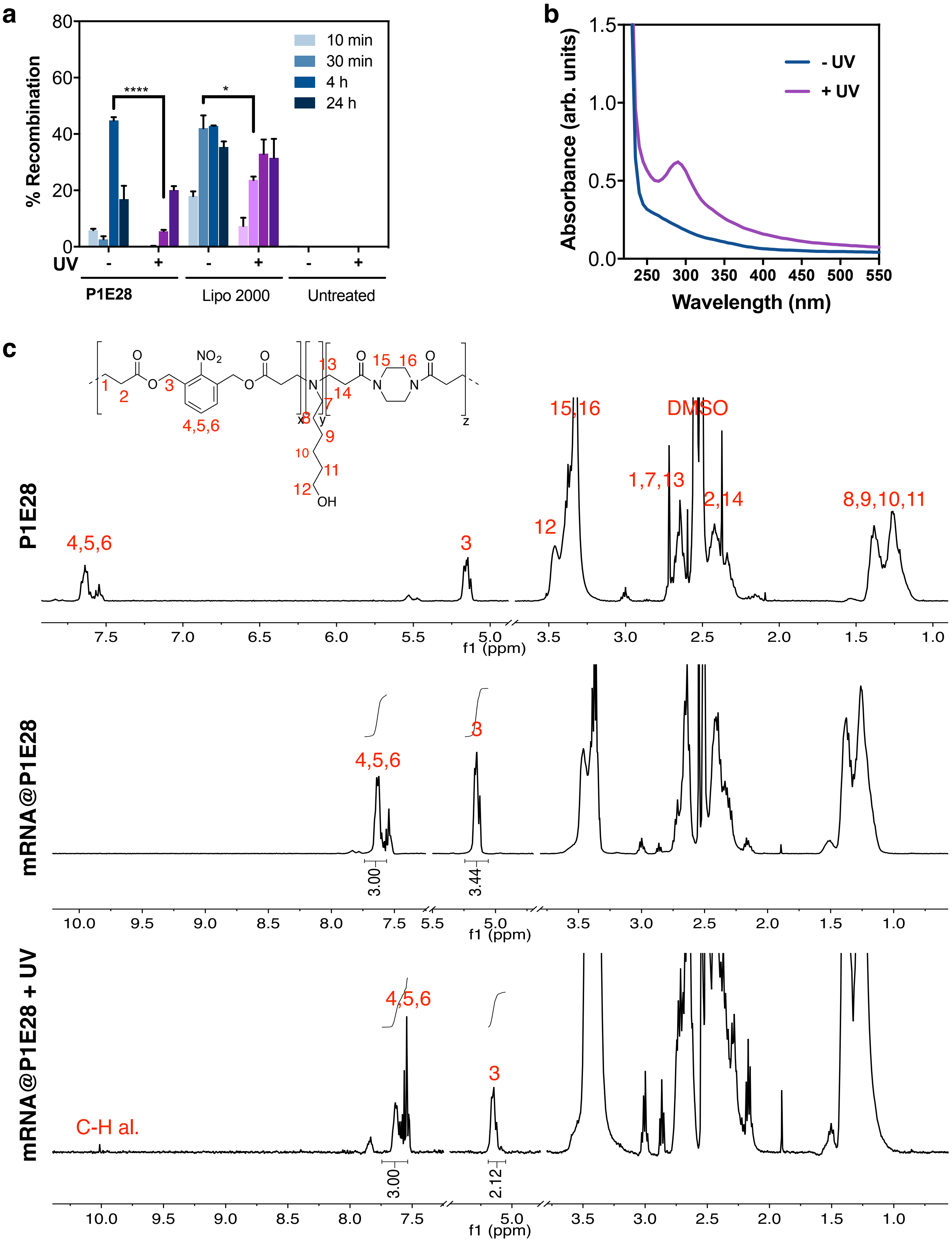

Figure S6. Characterization of polymer response to UV radiation. (a) Transfection efficiency of P1E28 was compared with Lipofectamine® 2000 at different time points, either in the presence or absence of UV radiation (λ = 365 nm, 10 mW cm^{-2}, 10 min). Two-way ANOVA with *post hoc* Sidak multiple comparisons test was performed: (*), $p < 0.05$; (****), $p < 0.0001$. Data are expressed as mean ± SEM (n = 3-5). **(b)** UV/Vis spectroscopy demonstrates polymer degradation after UV irradiation after the emergence of an absorption peak around 290 nm, owing to the generation of the nitrobenzene group. **(c)** ^{1}H NMR spectra of P1E28 alone (in DMSO-d$_6$) or complexed with Cre mRNA (in D_2O) before and UV irradiation. Highlighted peaks assigned NMR spectra to chemical structure of P1E28. Negligible proton signals describing the presence of acrylates (5-7 ppm) suggest that the polymer was effectively capped by terminal amines. Peak integration at 5.2 ppm (assigned to protons bound to the α carbon atom in the alkoxy group of ester bonds) was normalized by the peak at 7.6 ppm (corresponding to 3 protons in the aromatic group). UV irradiation decreased the peak at 5.2 ppm by 38%, while a small peak at 10 ppm emerged, which can be attributed to the generation of aldehydes, which could be reactive towards hydroxyl groups in mRNA.

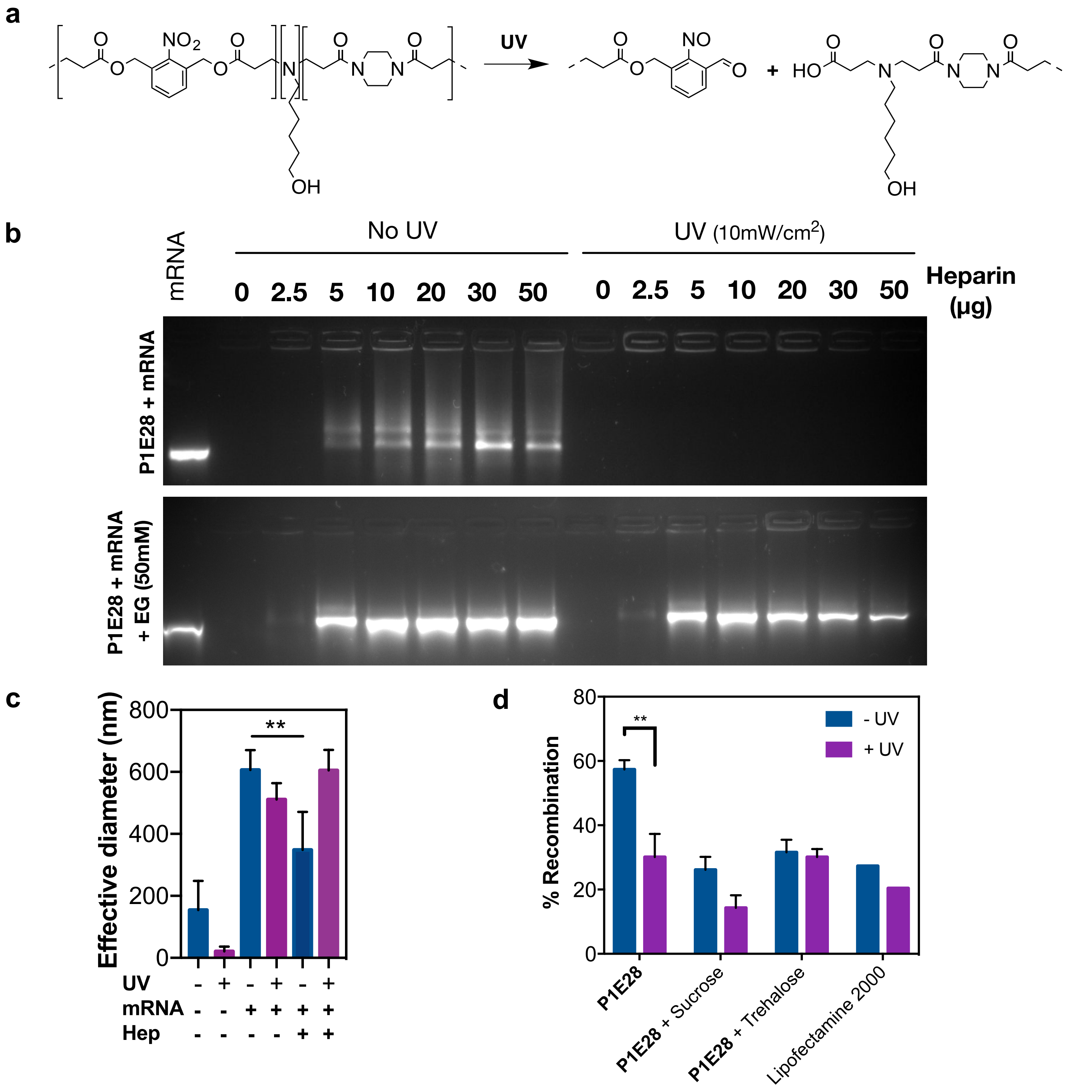

Figure S7. Characterization of polyplex stability after UV exposure. (a) Proposed mechanism of UV-induced photocleavage of P1E28. **(b)** Heparin replacement assay of P1E28 complexed with Cre mRNA was performed before and after UV exposure. Lack of mRNA release from polyplexes exposed to UV radiation led us to test the effect of aldehyde protecting groups such as ethylene glycol (EG), which constituted acetals with the reactive aldehydes and restored polyplex dissociation after UV exposure, without affecting particle stability. **(c)** Hydrodynamic diameter of P1E28 polyplexes in the presence or absence of UV and/or heparin was measured by DLS. While heparin destabilized P1E28 polyplexes by decreasing the average hydrodynamic diameter from 606.6 ± 63.6 nm to 348.1 ± 122.7 nm (p = 0.0187), UV exposure rendered the polyplexes insensitive to heparin (p = 0.5058). Data are expressed as mean ± SEM (n = 3-4). **(d)** Non-reducing sugars such as trehalose and sucrose are widely used to protect nucleic acids during lyophilization for long-term storage, and could mimic the effects of EG. These sugars rendered P1E28 insensitive to UV radiation. Data are expressed as mean ± SEM (n = 3). In **(c)** and **(d)**, two-way ANOVA with *post hoc* Sidak multiple comparisons test was performed: (**), $p < 0.01$.

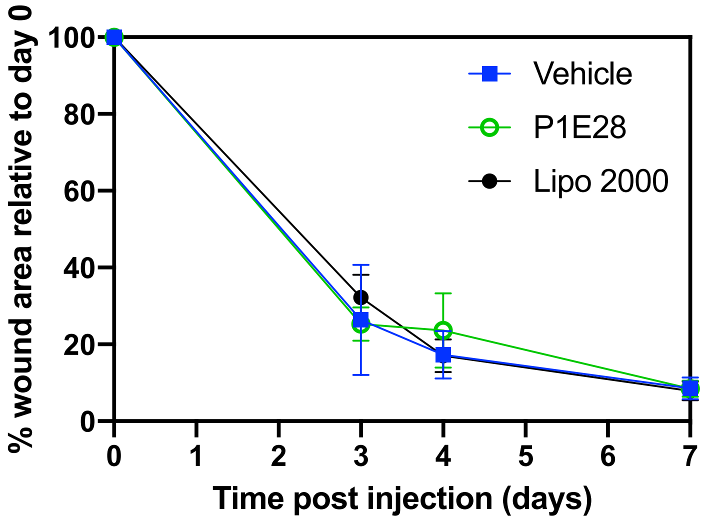

Figure S8. Wound healing process over time. Wound closure was quantified by comparing the reduction of wound area over time. Data are expressed as mean ± SEM (n = 2-6).

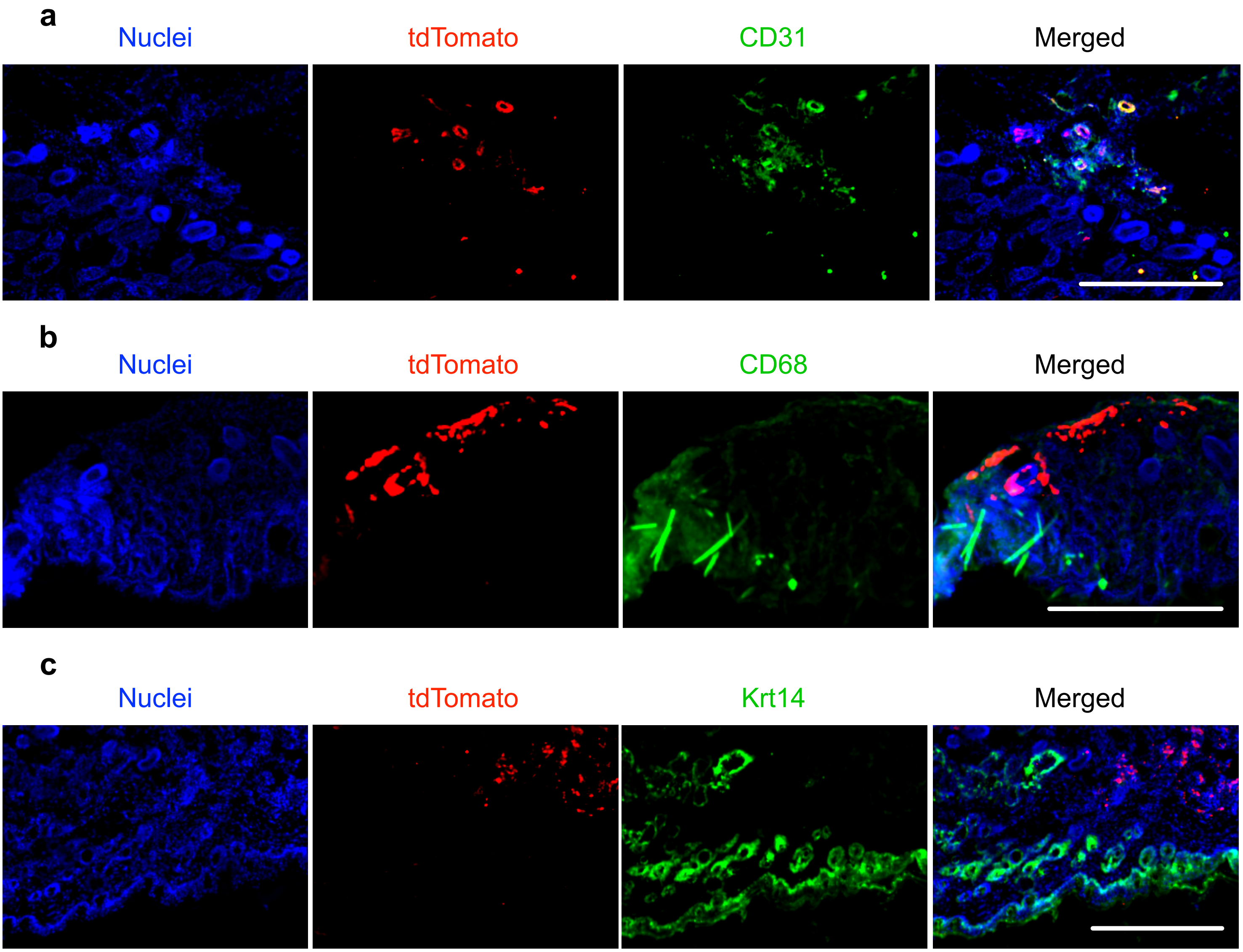

Figure S9. Immunohistochemical staining of skin sections. Representative fluorescence microscopy images monitoring co-localization of tdTomato signal with **(a)** CD31, **(b)** CD68, and **(c)** keratin 14 enabled the identification of transfected endothelial cells, macrophages, and keratinocytes, respectively. Scale bars = 50 μm.

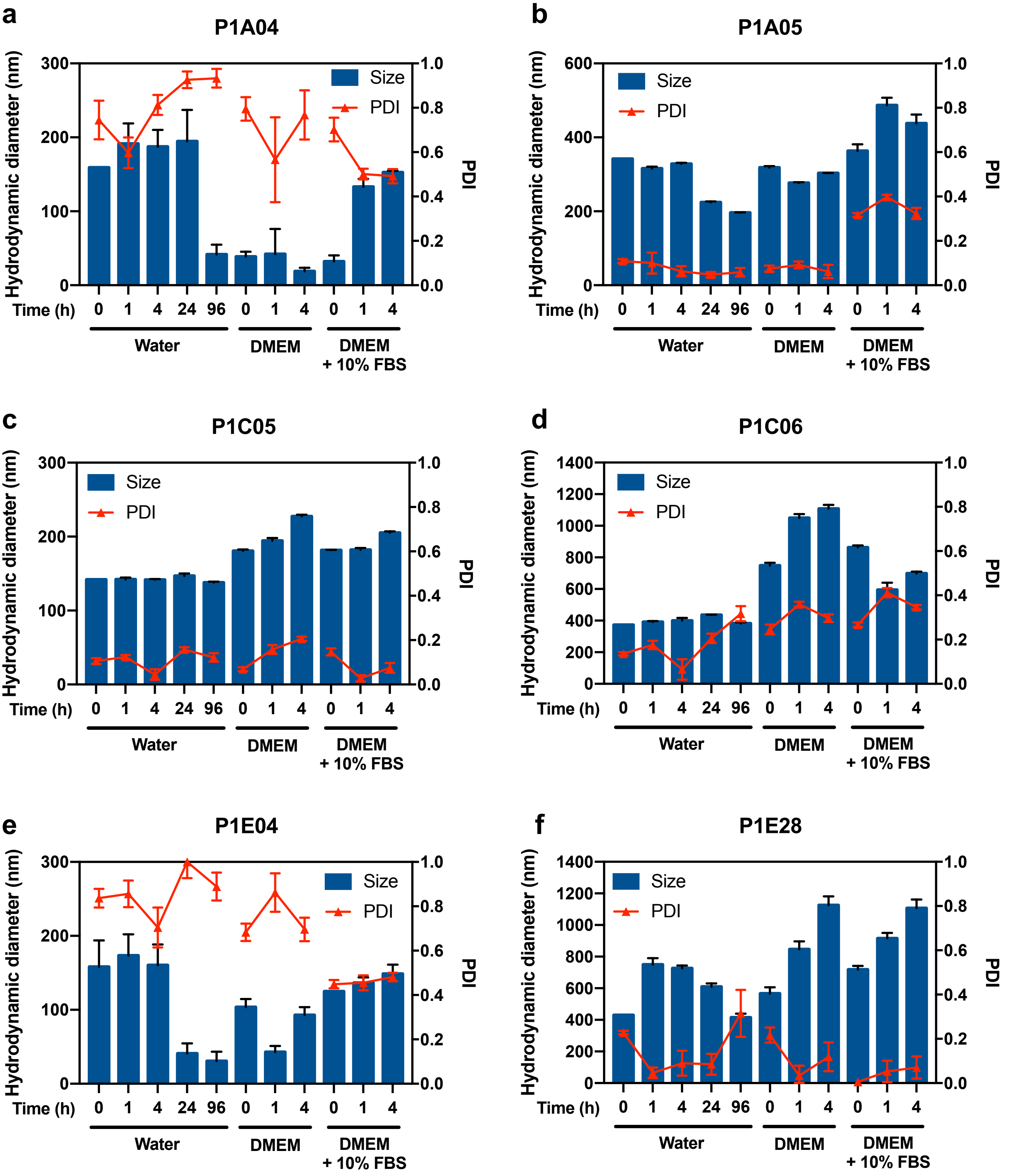

Figure S10. Polyplex stability in water and cell culture medium. Purified polymers were complexed with Cre mRNA at the optimized mRNA:polymer mass ratios and diluted in water or cell culture medium (DMEM with or without 10% FBS). Polyplex stability (hydrodynamic diameter and polydispersity index, PDI) was measured by DLS in water (for 96 h) and cell culture medium (for 4 h) for lead candidates **(a)** P1A04, **(b)** P1A05, **(c)** P1C05, **(d)** P1C06, **(e)** P1E04 and **(f)** P1E28. Data are expressed as mean ± SEM (n = 5).

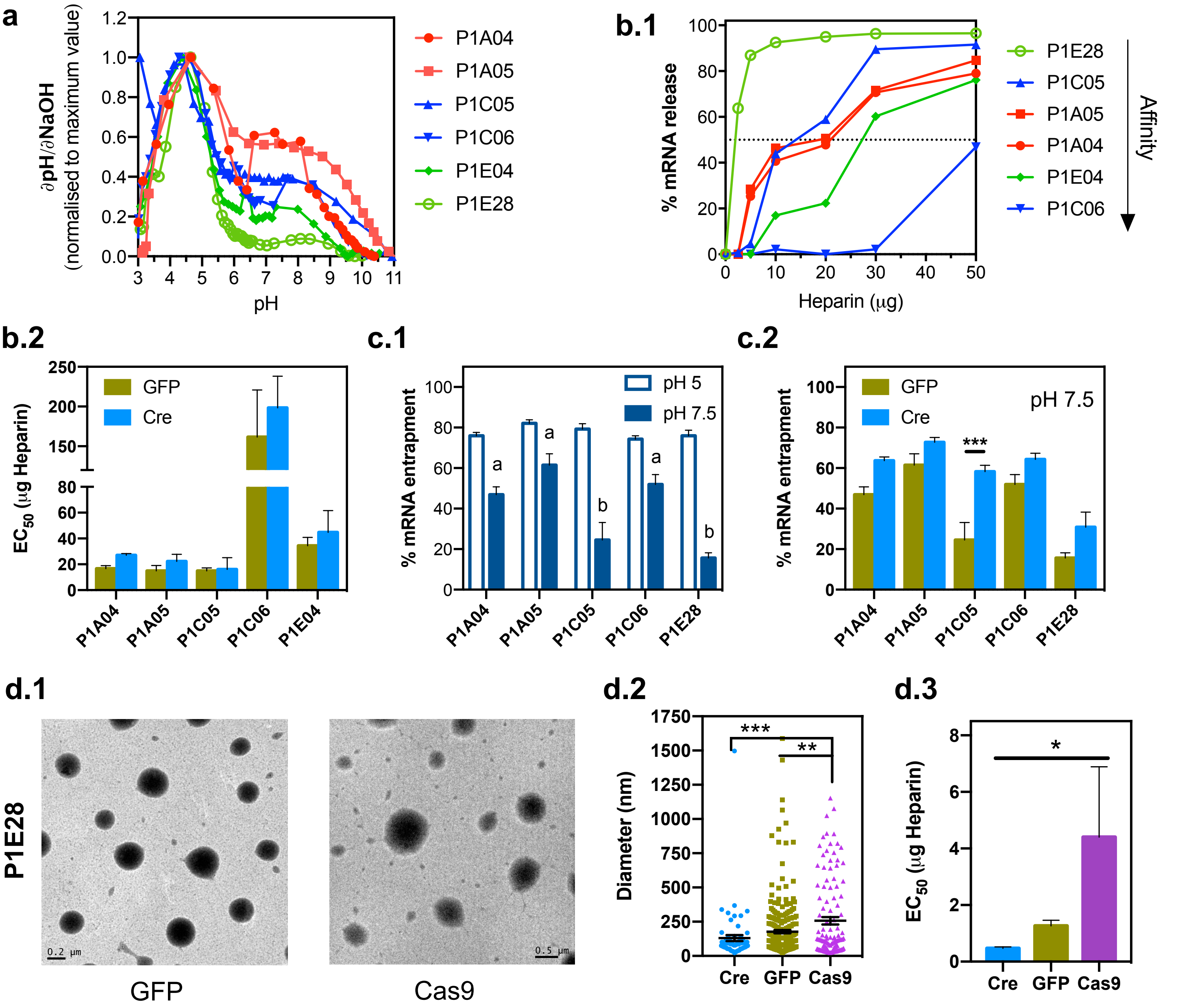

Figure S11. Influence of mRNA on polyplex physicochemical properties. (a) Normalized buffering capacity of each polymer was determined from the titration curves in **Figure 5c**. All polymers showed a clear effective pKa between pH 4-5, with weaker ionization steps at more basic pH (7-9). **(b)** Binding affinity of polymers complexed with GFP mRNA was estimated by heparin replacement assay, (b.1) showing similar profiles to those obtained with Cre mRNA (**Figure 5b**). (b.2) Estimated amount of heparin required to release 50% of complexed mRNA (EC50) was not significantly different between GFP and Cre mRNA ($p > 0.05$), after performing a two-way ANOVA with *post hoc* Tukey's multiple comparisons test. Results are expressed as mean ± SEM (n = 2-3). **(c)** Complexation efficiency of GFP mRNA was assessed by SYBR Gold labelling, (c.1) evidencing a similar pH-dependent behavior for all polymers. (c.2) Binding affinity toward GFP mRNA was compared with Cre mRNA (**Figure 5a**). Although the selected polymers seemed to complex GFP mRNA less efficiently than Cre mRNA at pH 7.5, this was only significant for P1C05. Results are expressed as mean ± SEM (n = 3). Two-way ANOVA was performed with *post hoc* Tukey's multiple comparisons test: (***), $p < 0.001$. In (c.1), statistically significant differences between groups are indicated by different letters ($p < 0.05$). **(d)** P1E28 was complexed with GFP mRNA or Cas9 mRNA + GFP sgRNA for structural characterization. (d.1) Representative TEM images and (d.2) size distribution of P1E28 complexed with different mRNAs illustrated larger polyplex size when the polymer is complexed with Cas9 mRNA. Bars in (d.2) correspond to mean ± SEM of >100 measured NPs: Cre = 130.9 ± 22.2 nm; GFP = 176.7 ± 11.9 nm; Cas9 = 256.8 ± 26.7 nm. One-way ANOVA test was performed with *post hoc* Tukey's multiple comparisons test: (**), $p < 0.01$; (***), $p < 0.001$. (d.3) Binding affinity determined by heparin replacement assay showed significantly higher affinity of P1E28 toward Cas9 mRNA and GFP sgRNA. Results are expressed as mean ± SEM (n = 2-3). A Kruskal-Wallis test was performed with *post hoc* Dunn's multiple comparisons test: (*), $p < 0.05$.

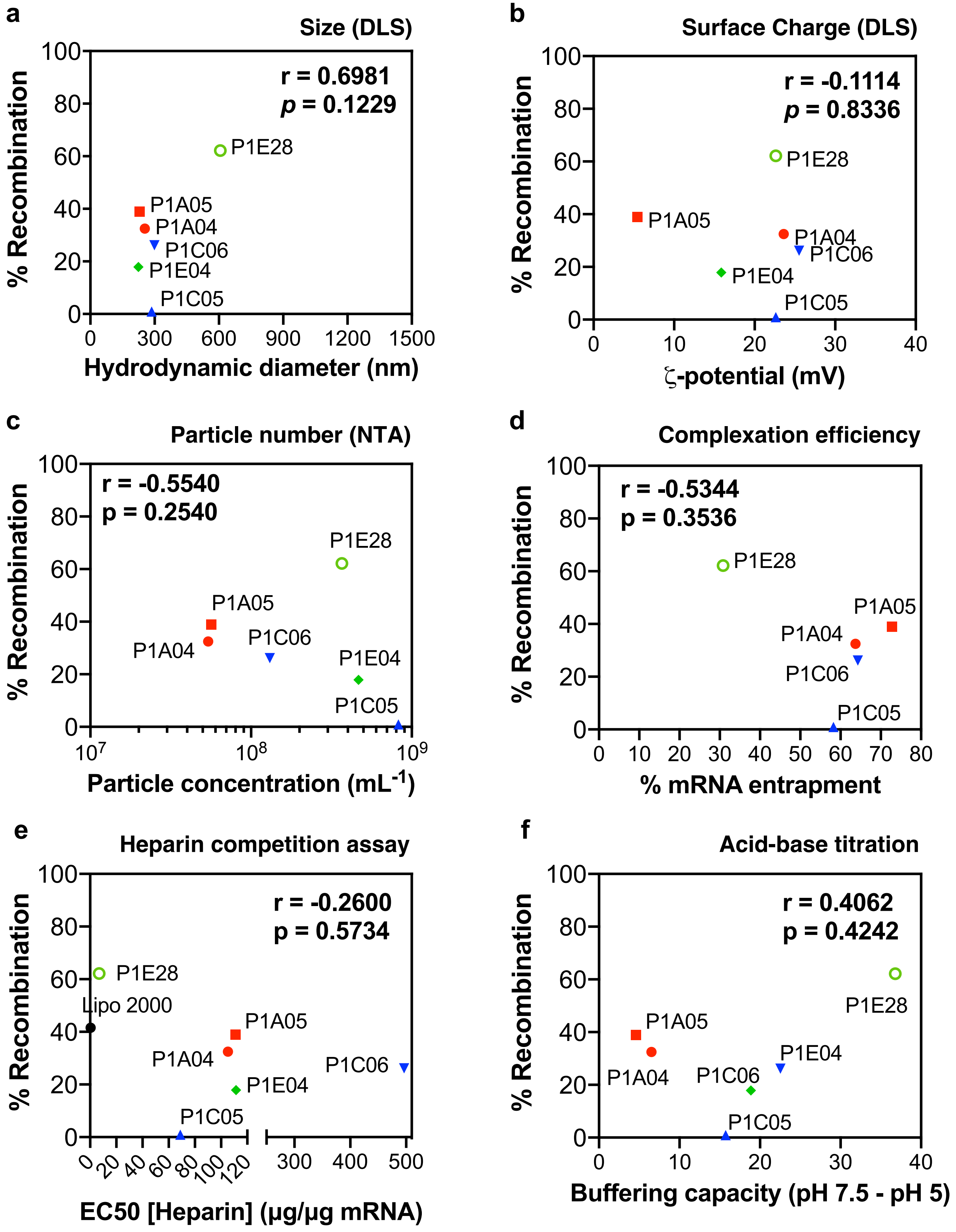

Figure S12. Lack of correlation between transfection efficiency and physicochemical properties of polyplexes. Data points represent the mean values of: **(a)** hydrodynamic diameter, and **(b)** ζ-potential, obtained by DLS; **(c)** particle number obtained by NTA; **(d)** complexation efficiency determined by SYBR Gold fluorescence assay; **(e)** binding affinity, represented by the dose of heparin required to induce 50% of mRNA release from polyplexes (EC50); **(f)** buffering capacity corresponding to the amount of NaOH required to increase solution pH from 5 to 7.5 (in µmol mg^{-1} polymer). Pearson correlation test showed no significant association of these individual parameters with transfection efficiency ($p > 0.05$).

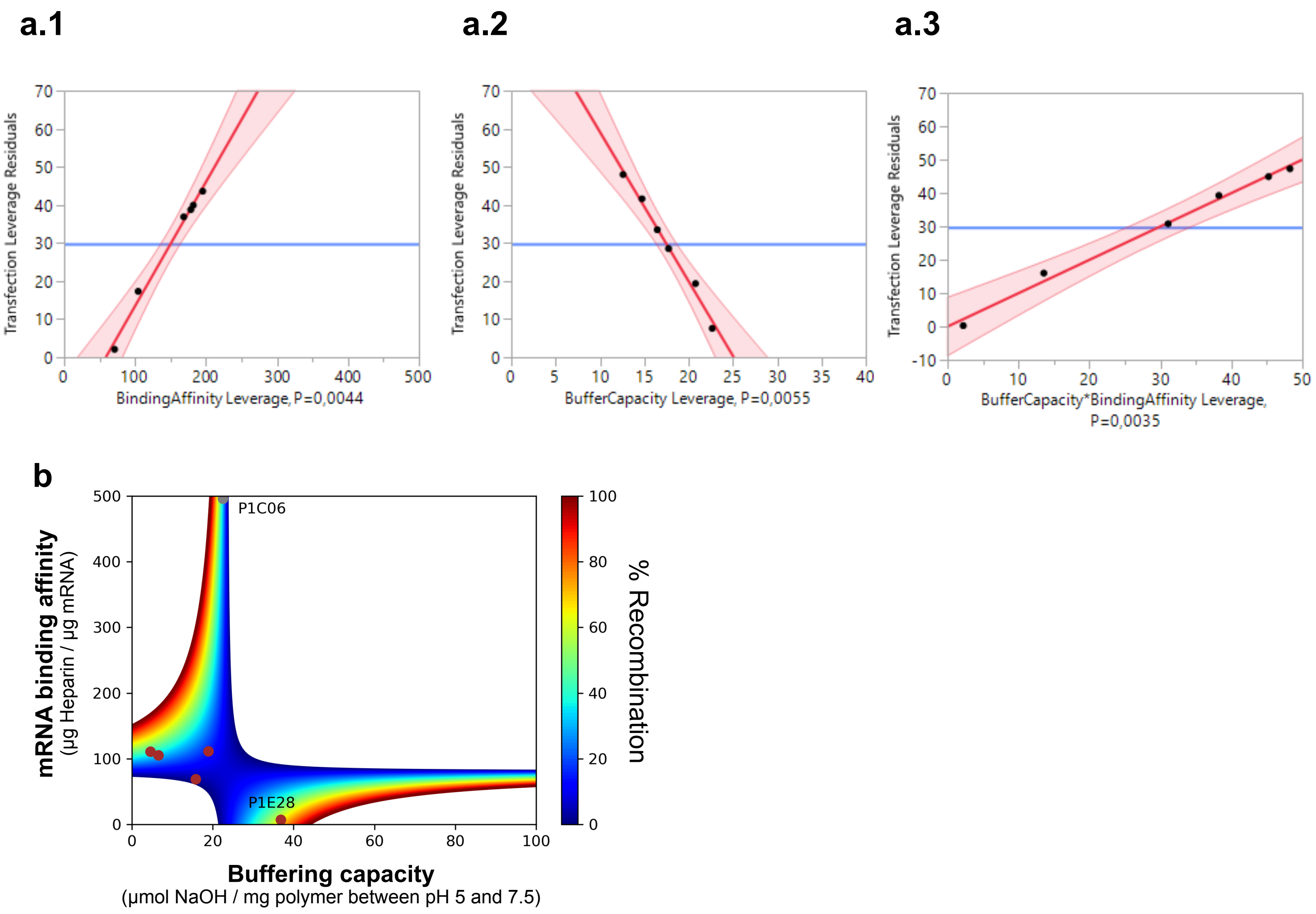

Figure S13. Multiple linear regression of polymer characteristics in transfection efficiency. (a) Transfection efficiency of the selected polymers was correlated simultaneously with their (a.1) mRNA binding affinity and (a.2) buffering capacity. Data were fitted to a standard least squares model, set to effect leverage. (a.3) First-order interactions between these parameters were also considered, given that P1E28 exhibited the highest buffering capacity, which is associated with successful endosomal release, and the lowest mRNA binding affinity, comparable to Lipofectamine® 2000 (**Figure 5b-c**). Both parameters were shown to interact negatively with each other and affect transfection efficiency (p = 0.0035). **(b)** Predicted model corresponding to **Figure 5d.2**, highlighting P1E28 and P1C06, which was not plotted in the main figure because it exhibited an exceptionally high mRNA binding affinity. For fixed transfection efficiency values ranging from 0 to 100, the generated model predicted that high biological activity could be attained by designing polymers with: i) low mRNA binding affinity (EC50 < 100 μg heparin μg^{-1} mRNA) and high buffering capacity (> 30 μmol NaOH mg^{-1} polymer); ii) high mRNA binding affinity (EC50 > 100 μg heparin μg^{-1} mRNA) and low buffering capacity (< 20 μmol NaOH mg^{-1} polymer).

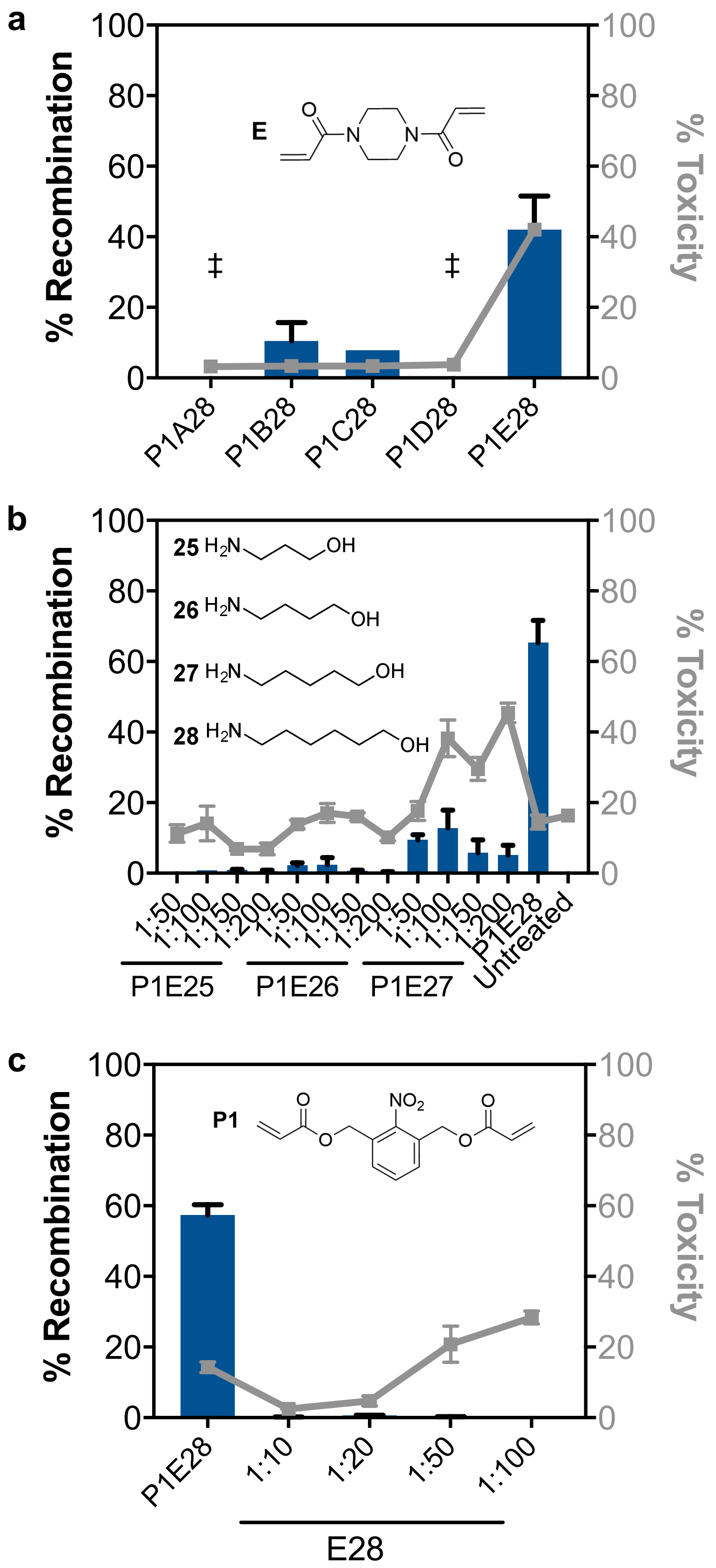

Figure S14. Comparison of transfection efficiency among polymers with similar chemical features. (a) Cre mRNA was complexed with non-purified polymers sharing the same amine 28, resulting in negligible activity compared to P1E28. Some polyplexes exhibited lower recombination rates than naked mRNA (‡). **(b)** Purified P1E28 transfected Cre mRNA more effectively than other similar polymers, sharing the same bisacrylamide and diacrylate moieties but varying amine group by the length of the alkyl chain. Although purified polymer doses were adjusted with the aim of optimising transfection efficiency, none of the tested polymers could outperform P1E28. **(c)** Transfection efficiency of P1E28 required the presence of the diacrylate moiety P1. Data are expressed as mean ± SEM (n = 3).

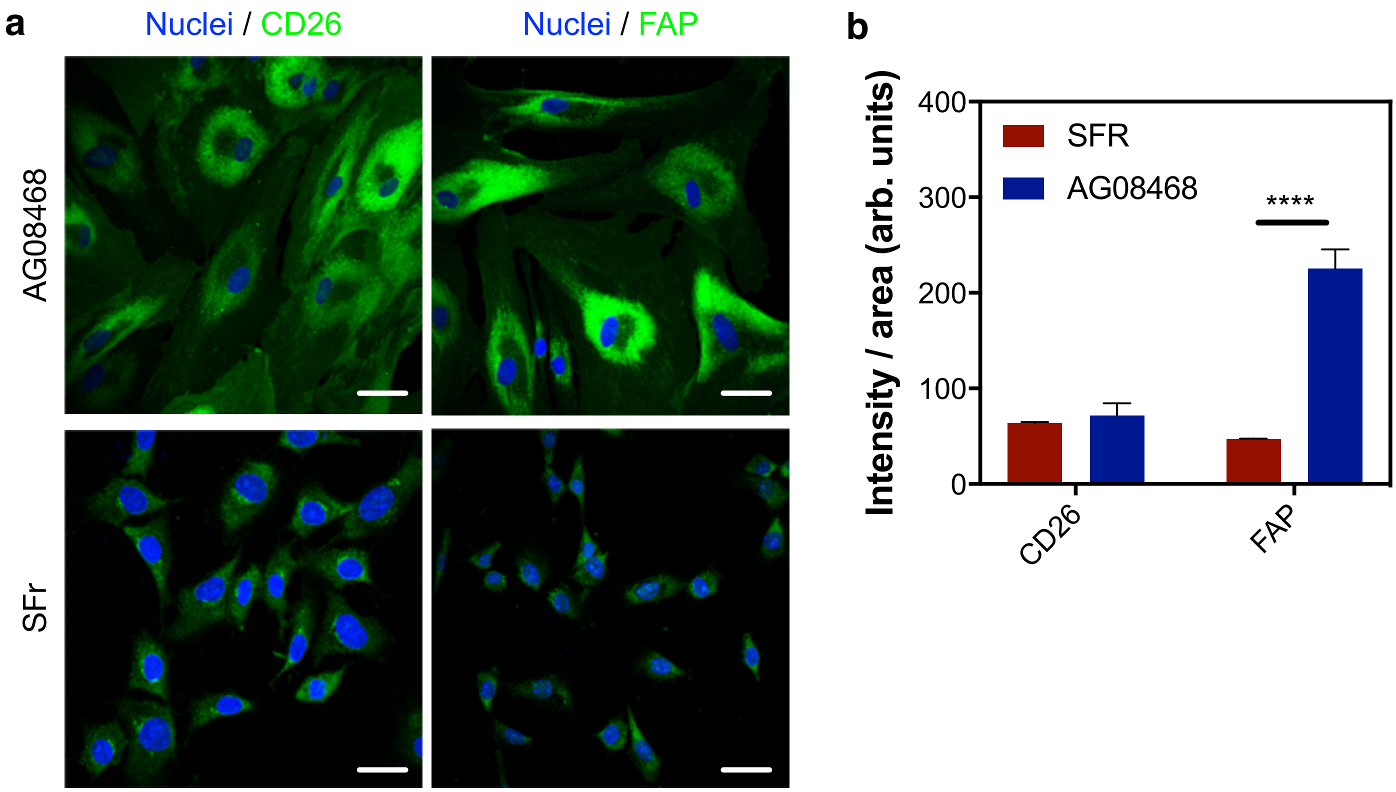

Figure S15. Characterization of CD26/DPP4 family receptors in fibroblasts. (a) Representative immunofluorescence images of human dermal fibroblasts (AG08468) and mouse reporter fibroblast model used for screening the polymer library (SFr) stained for CD26 (DPP4) and FAP. Scale bars = 30 µm. **(b)** Total fluorescence intensity of each protein was normalized by cell area in each field. Two-way ANOVA with *post hoc* Sidak multiple comparisons test was performed: (****), $p < 0.0001$. Data are expressed as mean ± SEM (n = 5 images obtained from 2 independent measurements per condition).

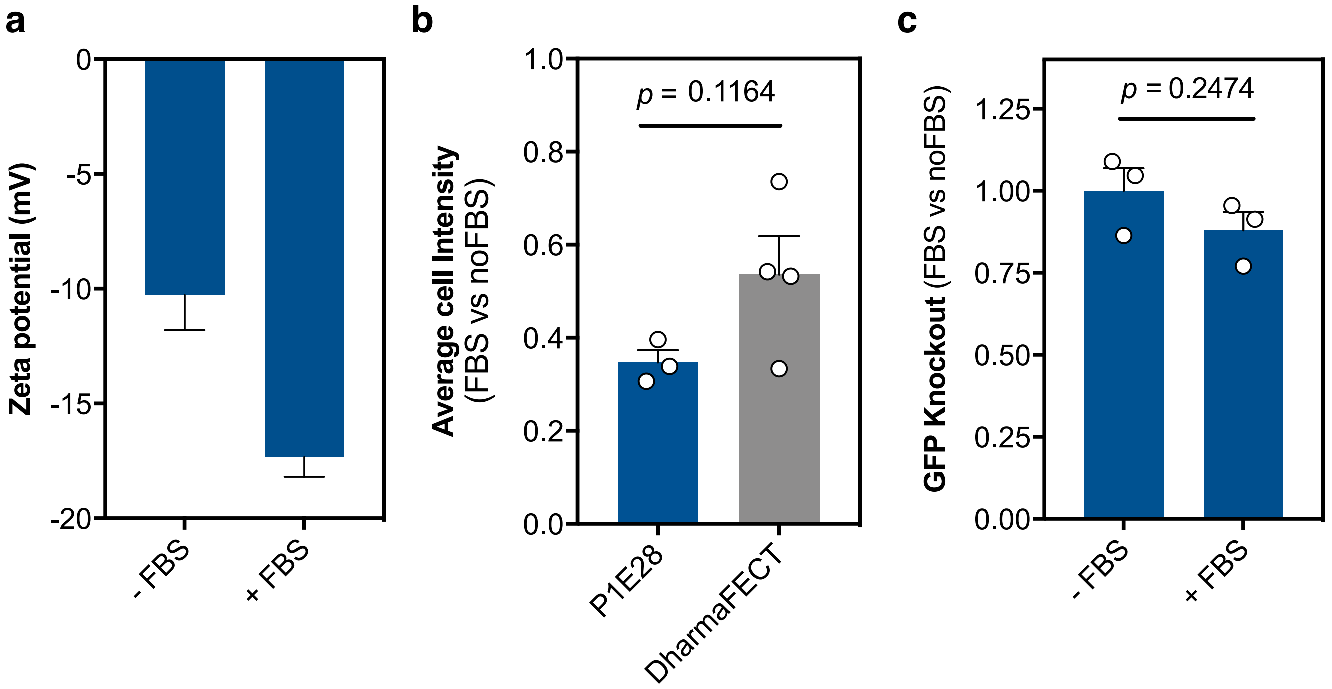

Figure S16. Influence of serum proteins in cellular uptake and activity of P1E28 complexed with Cas9 mRNA. (a) Zeta potential of P1E28 complexed with Cas9 mRNA and sgRNA after incubation for 4 h in cell culture medium in the absence (- FBS) or presence of FBS (+ FBS). Polyplexes were purified by centrifugation and resuspended in KCl (1 mM) before measurement. Data are expressed as mean ± SEM (n = 3). **(b)** Internalization of P1E28 complexed with Cas9 mRNA in SFr cells was compared to the commercial agent DharmaFECT Duo using an ATTO550-labelled sgRNA, after 4 h of incubation in the presence (FBS) or absence of FBS (noFBS). Total fluorescence intensity of ATTO550 was normalized by the number of cells in each field. Results of each treatment were normalized to uptake in medium without FBS (standard experimental conditions). Data are expressed as mean ± SEM (n = 3-4 replicates obtained from 2 independent experiments; each replicate corresponds to the average of 10 images). No statistical significance was obtained after performing an unpaired two-tailed t-test (p = 0.1164). **(c)** Biological activity of P1E28 complexed with Cas9 mRNA and sgRNA targeting GFP was evaluated in SFr-GFP cells, after 4 h of incubation in the absence (- FBS) or presence of FBS (+ FBS). Gene editing was characterized by the knockout of GFP, resulting in a reduced fluorescence. GFP knockout was evaluated by flow cytometry 3 days after incubation. Results of each treatment were normalized to uptake in medium without FBS (standard experimental conditions). Data are expressed as mean ± SEM (n = 3). No statistical significance was obtained after performing an unpaired two-tailed t-test (p = 0.2474).
